# Polymorphisms in genes involved in the absorption, distribution, metabolism, and excretion of drugs in the Kazakhs of Kazakhstan

**DOI:** 10.1186/s12863-016-0329-x

**Published:** 2016-01-19

**Authors:** Aisha N. Iskakova, Aliya A. Romanova, Akbota M. Aitkulova, Nurgul S. Sikhayeva, Elena V. Zholdybayeva, Erlan M. Ramanculov

**Affiliations:** National Scientific Laboratory of Biotechnology, National Center for Biotechnology, Almaty, Kazakhstan; Biology and Biotechnology Department, Al-Farabi Kazakh National University, Almaty, Kazakhstan; Faculty of Natural Sciences, L.N, Gumilyov Eurasian National University, Astana, Kazakhstan; School of Science and Technology Nazarbayev University, Astana, Kazakhstan

**Keywords:** Kazakhstan, Single nucleotide polymorphism, Adsorption, Distribution, Metabolism, Excretion, OpenArray

## Abstract

**Background:**

Studies of genes involved in the absorption, distribution, metabolism, and excretion (ADME) of drugs are crucial to the development of therapeutics in clinical medicine. Such data provide information that may improve our understanding of individual differences in sensitivity or resistance to certain drugs, thereby helping to avoid adverse drug reactions (ADRs) in patients and improve the quality of therapies. Here, we aimed to analyse single nucleotide polymorphisms (SNPs) involved in the ADME of multiple drugs in Kazakhs from Kazakhstan.

**Results:**

A total of 158 SNPs involved in the ADME of various drugs were studied. We analysed 320 Kazakh DNA samples using OpenArray genotyping. Of the 158 SNPs, 75 were not found in heterozygous or homozygous variants. Comparative analysis among Kazakhs and world populations showed a fairly high percentage of population differentiation.

**Conclusion:**

These results provide further information for pharmacogenetic databases and may contribute to the development of personalized approaches and safer therapies for the Kazakh population. Moreover, these data provide insights into the different racial groups that may have contributed to the Kazakh population.

**Electronic supplementary material:**

The online version of this article (doi:10.1186/s12863-016-0329-x) contains supplementary material, which is available to authorized users.

## Background

Current pharmacogenetic research includes the study of genes involved in the absorption, distribution, metabolism, and excretion (ADME) of drugs. These data may help clinicians and researchers to understand individual differences in sensitivity or resistance to certain drugs, thereby avoiding adverse drug reactions (ADRs) in patients and improving the quality of therapies. Thus, pharmacogenetic research has great practical value in the development of personalised medicine. Moreover, pharmacogenetic studies contribute to our understanding of population genetics because the frequencies of certain allelic variants may differ depending on the population. The people of Central Asia are poorly understood from a population genetic standpoint. However, studies in this field are on-going; the Kazakh population has been studied by both domestic and foreign scientists [1–4].

Kazakhs, one of the Turkic peoples of Central Asia, are the main population of Kazakhstan. According to the Committee on Statistics of the Republic of Kazakhstan, about 11 million Kazakhs live in Kazakhstan, and about 3.5 million Kazakhs live in regions neighbouring Kazakhstan and in other regions (China, Russia, Uzbekistan, Turkmenistan, Kyrgyzstan, west Mongolia, and Turkey) [5]. Kazakhs residing in the territory of Kazakhstan have an internal division into three large groups, Elder or Senior (Uly), Middle or Medium (Orta), and Lesser or Junior (Kishi) Zhuzes (or Hordes); historically, these three groups had demarcated territories. Additionally, there were several tribes in each Zhuz [6]. Every Kazakh knows to which tribe and Zhuz he or she belongs, and representatives of the same tribe are considered relatives as they have descended from a common ancestor. Therefore, according to the seven generations law, marriage between members of the same tribe is only possible after seven generations from a common ancestor [7].

Many genes associated with the ADME of drugs have been identified. A team including representatives of the pharmaceutical industry and an academic centre developed a core list of 32 ADME genes, which includes 184 markers that can be used to screen patients in clinical trials. These data are available on the PharmaADME website (http://pharmaadme.org/).

In this study, we aimed to analyse single nucleotide polymorphisms (SNPs) involved in the ADME of multiple drugs in Kazakhs from Kazakhstan using an OpenArray PGx Panel derived from the PharmaADME Core Marker List.

## Results

### Allele and genotype frequency analysis

Allele and genotype frequency data were obtained for 158 SNPs (Additional file 1). Seventy-five out of the 158 SNPs included in this study were not found in heterozygous or homozygous variants (Additional file 2). The allele and genotype frequencies of the remaining 83 SNPs are summarized in Table 1.

**Table 1:** Allele frequency and genotype distribution in the Kazakh population (^а^ number of chromosomes; ^b^ number of alleles)

	#	Assay name	RS	Number of samples	Hardy-Weinberg equilibrium *p*-value	Allele	n^a^	Frequency	Genotype	n^b^	Frequency
drug transporters		ATP-binding cassette									
	1	ABCB1_C3435ntT	rs1045642	280	0.39184	A	257	0.46	AA	55	0.20
						G	303	0.54	AG	147	0.53
									GG	78	0.8
	2	ABCB1_T1236ntC	rs1128503	278	0.72281	A	292	0.53	AA	75	0.27
						G	264	0.47	AG	142	0.51
									GG	61	0.22
	3	ABCB1_2677nt G > T	rs2032582	275	0.03713	C	308	0.56	СС	95	0.35
						A	242	0.44	CA	118	0.43
									AA	62	0.23
	4	ABCB1_2677nt G > A	rs2032582	240	0	C	409	0.85	СС	189	0.79
						T	71	0.15	СT	31	0.13
									TT	20	0.08
	5	ABCC2_V417I	rs2273697	279	0.03662	G	472	0.85	GG	195	0.70
						A	86	0.15	GA	82	0.29
									AA	2	0.01
	6	ABCC2_I1324I	rs3740066	279	0.32311	C	388	0.70	СС	131	0.47
						T	170	0.30	СT	126	0.45
									TT	22	0.08
	7	ABCC2_-24C > T	rs717620	292	0.36682	C	467	0.80	СС	184	0.63
						T	117	0.20	СT	99	0.34
									TT	9	0.03
	8	ABCG2_ 421 nt C > A	rs2231142	281	1	G	476	0.85	GG	201	0.72
						T	86	0.15	GT	74	0.26
									TT	6	0.02
		Solute carrier family 15 (H+/peptide transporter)									
	9	SLC15A2_P409S	rs1143671	288	1	T	315	0.55	TT	86	0.30
						C	261	0.45	TC	143	0.50
									CC	59	0.20
	10	SLC15A2_R509K	rs1143672	258	0.5314	A	282	0.55	AA	74	0.29
						G	234	0.45	AG	134	0.52
									GG	50	0.19
	11	SLC15A2_A284A	rs2293616	287	0.5535	A	302	0.53	AA	82	0.29
						G	272	0.47	AG	138	0.48
									GG	67	0.23
	12	SLC15A2_L350F	rs2257212	277	0.90495	C	252	0.45	СС	58	0.21
						T	302	0.55	СT	136	0.49
									TT	83	0.30
		Solute carrier family 22 (organic cation transporter)									
	13	SLC22A1_420del3	rs72552763	280	0.43952	GAT	512	0.91	GAT/GAT	235	0.84
						del	48	0.09	GAT/del	42	0.15
									del/del	3	0.01
	14	SLC22A1_P283L	rs4646277	269	1	C	525	0.98	СС	256	0.95
						T	13	0.02	СT	13	0.05
									TT	0	0.00
	15	SLC22A1_P341L	rs2282143	242	0.36537	C	463	0.96	СС	222	0.92
						T	21	0.04	СT	19	0.08
									TT	1	0.00
	16	SLC22A1_M408V	rs628031	272	0.79314	G	351	0.65	GG	112	0.41
						A	193	0.35	GA	127	0.47
									AA	33	0.12
	17	SLC22A1_G465R	rs34059508	283	0.06258	G	557	0.98	GG	275	0.97
						A	9	0.02	GA	7	0.02
									AA	1	0.00
	18	SLC22A2_K432Q	rs8177517	276	1	T	544	0.99	TT	268	0.97
						G	8	0.01	TG	8	0.03
									GG	0	0.00
	19	SLC22A2_M165I	rs8177507	289	1	C	569	0.98	СС	280	0.97
						T	9	0.02	СT	9	0.03
									TT	0	0.00
	20	SLC22A2*4.1_S270A	rs316019	267	0.376	C	495	0.93	CC	228	0.85
						A	39	0.07	CA	39	0.15
									AA	0	0.00
		Solute carrier organic anion transporter family									
	21	SLCO1B1*1B_N130D	rs2306283	267	0.53112	G	307	0.57	GG	91	0.34
						A	227	0.43	GA	125	0.47
									AA	51	0.19
	22	SLCO1B1*5_V174A	rs4149056	243	0.14152	T	410	0.84	TT	176	0.72
						C	76	0.16	TC	58	0.24
									CC	9	0.04
	23	SLCO1B3_699G > A	rs7311358	265	0.13227	A	402	0.76	AA	157	0.59
						G	128	0.24	GA	88	0.33
									GG	20	0.08
	24	SLCO1B3_334T > G	rs4149117	254	0.85656	G	395	0.78	GG	154	0.61
						T	113	0.22	GT	87	0.34
									TT	13	0.05
	25	SLCO2B1*3_S486F	rs2306168	275	0.11608	C	461	0.84	СС	197	0.72
						T	89	0.16	СT	67	0.24
									TT	11	0.04
Phase II metabolizing enzymes		Glutathione S-transferase pi 1									
	26	GSTP1_V105I	rs1695	276	0.71558	C	437	0.79	AA	174	0.63
						T	115	0.21	AG	89	0.32
									GG	13	0.05
		N-acetyltransferase									
	27	NAT1_884A > G	rs55793712	294	1	A	584	0.99	AA	290	0.99
						G	4	0.01	AG	4	0.01
									GG	0	0.00
	28	NAT1*11A-C g.-344C > T	rs4986988	278	1	C	545	0.98	СС	267	0.96
						T	11	0.02	СT	11	0.04
									TT	0	0.00
	29	NAT1*14_560G > A	rs4986782	289	1	G	576	1.00	GG	287	0.99
						A	2	0.00	GA	2	0.01
									AA	0	0.00
	30	NAT2*5_341T > C	rs1801280	256	0.00661	C	136	0.27	СС	27	0.11
						T	376	0.73	СT	82	0.32
									TT	147	0.57
	31	NAT2*6_590G > A	rs1799930	269	0.62646	G	402	0.75	GG	148	0.55
						A	136	0.25	GA	106	0.39
									AA	15	0.06
	32	NAT2*7_857G > A	rs1799931	278	1	A	62	0.11	AA	3	0.01
						G	494	0.89	GA	56	0.20
									GG	219	0.79
	33	NAT2*11_481C > T	rs1799929	267	0.03425	C	398	0.75	СС	155	0.58
						T	136	0.25	СT	88	0.33
									TT	24	0.09
	34	NAT2*12_803A > G	rs1208	272	0.18999	A	409	0.75	AA	158	0.58
						G	135	0.25	AG	93	0.34
									GG	21	0.08
	35	NAT2*13_282C > T	rs1041983	293	0.13049	C	368	0.63	СС	109	0.37
						T	218	0.37	СT	150	0.51
									TT	34	0.12
		Thiopurine S-methyltransferase									
	36	TPMT*3B_460 G > A A154T	rs1800460	249	1	C	492	0.99	CC	243	0.98
						T	6	0.01	CT	6	0.02
									TT	0	0.00
	37	TPMT*3C_719A > G C240Y	rs1142345	242	1	T	477	0.99	TT	235	0.97
						C	7	0.01	TC	7	0.03
									CC	0	0.00
		UDP glucuronosyltransferase									
	38	UGT1A1*27_686C > A	rs35350960	289	1	C	577	1.00	СС	288	1.00
						A	1	0.00	CA	1	0.00
									AA	0	0.00
	39	UGT2B15*2_253G > T	rs1902023	255	0.61468	A	281	0.55	AA	75	0.29
						C	229	0.45	AC	131	0.51
									CC	49	0.19
	40	UGT1A1*6_211G > A	rs4148323	288	0.66077	G	485	0.84	GG	205	0.71
						A	91	0.16	GA	75	0.26
									AA	8	0.03
	41	UGT2B7*2b_-327G > A	rs7662029	286	0.72247	A	307	0.54	AA	84	0.29
						G	265	0.46	AG	139	0.49
									GG	63	0.22
	42	UGT1A1*60_-3263 T > G	rs4124874	274	0.52854	T	326	0.59	TT	94	0.34
						G	222	0.41	TG	138	0.50
									GG	42	0.15
	43	UGT2B7*2a_-161C > T	rs7668258	277	0.90334	T	295	0.53	TT	79	0.29
						C	259	0.47	TC	137	0.49
									CC	61	0.22
		Dihydropyrimidine dehydrogenase									
Phase I metabolizing enzymes	44	DPYD*2A_IVS14 + 1G > A	rs3918290	288	1	C	575	1.00	СС	287	1.00
						T	1	0.00	СT	1	0.00
									TT	0	0.00
	45	DPYD*7_delTACT	hCV32287186	279	1	ATGA	551	0.99	ATGA/ATGA	272	0.97
						del	7	0.01	ATGA/del	7	0.03
									del/del	0	0.00
	46	DPYD*9A_C29R	rs1801265	268	0.8213	A	450	0.84	AA	188	0.70
						G	86	0.16	AG	74	0.28
									GG	6	0.02
		Cytochrome P450									
	47	CYP1A1*2 g.2455A > G	rs1048943	279	0.20473	T	463	0.83	TT	195	0.70
						C	95	0.17	TC	73	0.26
									CC	11	0.04
	48	CYP1A1*4 g.2453C > A	rs1799814	282	0.17395	G	549	0.97	GG	268	0.95
						T	15	0.03	GT	13	0.05
									TT	1	0.00
	49	CYP1A1*9 g.2461C > T	rs41279188	294	1	G	587	1.00	GG	293	1.00
						A	1	0.00	GA	1	0.00
									AA	0	0.00
	50	CYP1A2*1C g.-3860G > A	rs2069514	259	0.01548	G	437	0.84	GG	190	0.73
						A	81	0.16	GA	57	0.22
									AA	12	0.05
	51	CYP1A2*1 F g.-163C > A	rs762551	297	0.60371	A	394	0.66	AA	133	0.45
						C	200	0.34	CA	128	0.43
									CC	36	0.12
	52	CYP1A2*1 K g.-729C > T	rs12720461	294	1	C	587	1.00	СС	293	1.00
						T	1	0.00	СT	1	0.00
									TT	0	0.00
	53	CYP2A6*2 g.1799 T > A	rs1801272	261	1	A	515	0.99	AA	254	0.97
						T	7	0.01	AT	7	0.03
									TT	0	0.00
	54	CYP2A6*9 g.-48 T > G	rs28399433	261	0.06641	A	460	0.88	AA	206	0.79
						C	62	0.12	AC	48	0.18
									CC	7	0.03
	55	CYP2B6*6 g.15631G > T	rs3745274	232	0.40492	G	339	0.73	GG	121	0.52
						T	125	0.27	GT	97	0.42
									TT	14	0.06
	56	CYP2B6*8 g.13072A > G	rs12721655	265	1	A	529	1.00	AA	264	1.00
						G	1	0.00	AG	1	0.00
									GG	0	0.00
	57	CYP2B6*16 g. 21011 T > C	rs28399499	299	1	T	583	0.97	TT	284	0.95
						C	15	0.03	TC	15	0.05
									CC	0	0.00
	58	CYP2C8*2 g.11054A > T	rs11572103	275	1	T	548	1.00	TT	273	0.99
						A	2	0.00	TA	2	0.01
									AA	0	0.00
	59	CYP2C8*3 g.30411A > G	rs10509681	278	1	T	530	0.95	TT	252	0.91
						C	26	0.05	TC	26	0.09
									CC	0	0.00
	60	CYP2C8*3 g.2130G > A	rs11572080	270	1	C	512	0.95	СС	242	0.90
						T	28	0.05	СT	28	0.10
									TT	0	0.00
	61	CYP2C8*4 g.11041C > G	rs1058930	282	1	G	557	0.99	GG	275	0.98
						C	7	0.01	GC	7	0.02
									CC	0	0.00
	62	CYP2C9*2 g.3608C > T	rs1799853	277	1	C	527	0.95	СС	250	0.90
						T	27	0.05	СT	27	0.10
									TT	0	0.00
	63	CYP2C9*3 g.42614A > C	rs1057910	247	0.37469	A	457	0.93	AA	210	0.85
						C	37	0.07	AC	37	0.15
									CC	0	0.00
	64	CYP2C9*12 g.50338C > T	rs9332239	282	1	C	535	0.95	СС	253	0.90
						T	29	0.05	СT	29	0.10
									TT	0	0.00
	65	CYP2C19	rs17878459	239	1	G	475	0.99	GG	236	0.99
						C	3	0.01	GC	3	0.01
									CC	0	0.00
	66	CYP2C19 g.99C > T	rs17885098	259	0.14454	C	50	0.10	СС	0	0.00
						T	468	0.90	СT	50	0.19
									TT	209	0.81
	67	CYP2C19*2 g.19154G > A (splicing defect)	rs4244285	265	0.28264	G	439	0.83	GG	179	0.68
						A	91	0.17	GA	81	0.31
									AA	5	0.02
	68	CYP2C19*2 g.80160C > T	rs3758580	283	0.3245	C	462	0.82	СС	191	0.67
						T	104	0.18	СT	80	0.28
									TT	12	0.04
	69	CYP2C19*3 g.17948G > A	rs4986893	278	1	G	533	0.96	GG	255	0.92
						A	23	0.04	GA	23	0.08
									AA	0	0.00
	70	CYP2C19*3B g.87313A > C	rs17886522	289	1	A	547	0.95	AA	258	0.89
						C	31	0.05	AC	31	0.11
									CC	0	0.00
	71	CYP2C19*8 g.12711 T > C	rs41291556	119	1	T	237	1.00	TT	118	0.99
						C	1	0.00	TC	1	0.01
									CC	0	0.00
	72	CYP2C9*11 g.42542C > T	rs28371685	277	1	C	553	1.00	СС	276	1.00
						T	1	0.00	СT	1	0.00
									TT	0	0.00
	73	CYP2C19*17 g.-806C > T	rs12248560	279	0.00451	C	494	0.89	СС	224	0.80
						T	64	0.11	СT	46	0.16
									TT	9	0.03
	74	CYP2D6*3 g.2549delA	rs35742686	280	1	T	545	0.97	T/T	265	0.95
						del	15	0.03	T/del	15	0.05
									del/del	0	0.00
	75	CYP2D6*4 g.1846G > A	rs3892097	290	0.42114	C	507	0.87	СС	223	0.77
						T	73	0.13	СT	61	0.21
									TT	6	0.02
	76	CYP3A5*5 g.12952 T > C	rs55965422	272	1	A	525	0.97	AA	253	0.93
						G	19	0.03	AG	19	0.07
									GG	0	0.00
	77	CYP2D6*7 g.2935A > C	rs5030867	264	1	T	527	1.00	TT	263	1.00
						G	1	0.00	TG	1	0.00
									GG	0	0.00
	78	CYP2D6*9 g.2613_2615delAGA	rs72549350	296	1	TCT	570	0.96	TCT/TCT	274	0.93
						del	22	0.04	TCT/del	22	0.07
									del/del	0	0.00
	79	CYP2D6*40 g.1863_1864ins(TTTCGCCCC)2	hCV32407240	292	1	-	577	0.99	−/−'	285	0.98
						ins	7	0.01	Ins/-	7	0.02
									Ins/Ins	0	0.00
	80	CYP2E1*2 g.1132G > A	rs72559710	290	1	G	577	0.99	GG	287	0.99
						A	3	0.01	GA	3	0.01
									AA	0	0.00
	81	CYP3A4*2 g.15713 T > C	rs55785340	273	1	A	540	0.99	AA	267	0.98
						G	6	0.01	AG	6	0.02
									GG	0	0.00
	82	CYP3A4*6 c.830_831insA	rs4646438	276	1	-	551	1.00	−/−	275	1.00
						insA	1	0.00	-/insA	1	0.00
									insA/insA	0	0.00
drug targets		Vitamin K epoxide reductase complex									
	83	VKORC1	rs8050894	268	0.43311	G	337	0.63	GG	109	0.41
						C	199	0.37	GC	119	0.44
									CC	40	0.15

The correspondence of the distributions of genotype frequencies to the Hardy-Weinberg equilibrium was assessed using exact tests with a modified version of the Markov-chain random walk algorithm [8] (*р* > 0.05). Seven SNPs of the 83 (i.e., rs1799929 [*p* = 0.03], rs2069514 [*p* = 0.02], rs1801280 [*p* = 0.01], rs12248560 [*p* = 0.00], rs2032582 G > A [*p* = 0.00], rs2032582 G > T [*p* = 0.04], and rs2273697 [*p* = 0.04]) were not in Hardy-Weinberg equilibrium.

### Comparative analysis of allele frequencies

A comparative analysis of the allele frequency between the Kazakh samples analysed here and HapMap published data from 11 populations worldwide was carried out (Table 2). A comparative analysis was performed for those SNPs found in the Kazakh population in heterozygous or homozygous variants. hCV32287186, hCV32407240, rs2069514, rs17885098, rs72552763, rs35742686, rs72549350, rs5030867, rs55965422, rs11572080, rs4986893, rs72559710, rs41291556, rs55793712, rs35350960, rs55785340, rs2032582, rs3892097, rs17878459, rs28399433, rs34059508, rs41279188, rs4646438, rs17886522, rs12721655, and rs1902023 frequency data are missing in the HapMap database; therefore, these SNPs were not analysed, and comparative analysis was carried out for 56 SNPs. Exact tests of population differentiation with a significance level of 0.05 were used [9]. No statistically significant differences in the frequencies of rs8177507, rs3740066, rs4986988, rs1799930, rs4646277, or rs1801272 genotypes were found with any population (*p* > 0.05).

**Table 2:** A comparative analysis of the allele frequency between Kazakh population (our data) and world’s populations (HapMap data)

	#	Assay name	RS	Exact test of population differentiation (*P* value)										
				ASW	CEU	CHB	CHD	GIH	JPT	LWK	MEX	MKK	TSI	YRI
drug transporters		ATP-binding cassette												
	1	ABCB1_C3435ntT	rs1045642	0.00 + −0.00	0.01 + −0.00	0.07 + −0.00	0.06 + −0.0038	0.00 + −0.00	0.80 + −0.01		0.94 + −0.00	0.00 + −0.00	0.70 + −0.01	0.00 + −0.00
	2	ABCB1_T1236ntC	rs1128503	0.00 + −0.00	0.08 + −0.01	0.00 + −0.00	0.00 + −0.00	0.25 + −0.01	0.35 + −0.01	0.00 + −0.00	0.39 + −0.01	0.00 + −0.00	0.06 + −0.00	0.00 + −0.00
	3	ABCB1_2677nt G > T	rs2032582	0.00 + −0.00	0.01 + −0.00	0.69 + −0.01	0.01 + −0.00	0.03 + −0.00	0.99 + −0.00	0.00 + −0.00	0.02 + −0.00	0.00 + −0.00	0.02 + −0.00	
	4	ABCC2_V417I	rs2273697	0.08 + −0.00	0.00 + −0.00	0.07 + −0.0057	0.13 + −0.01	0.00 + −0.00	0.40 + −0.01	0.14 + −0.00	0.26 + −0.01	0.00 + −0.00	0.00 + −0.00	0.02 + −0.00
	5	ABCC2_I1324I	rs3740066		0.58 + −0.01	0.78 + −0.0050			0.69 + −0.01					0.82 + −0.00
	6	ABCC2_-24C > T	rs717620	0.00 + −0.00	0.54 + −0.01	0.87 + −0.0056	0.67 + −0.01	0.00 + −0.00	0.55 + −0.01	0.00 + −0.00	0.41 + −0.01	0.00 + −0.00	1.00 + −0.00	0.00 + −0.00
	7	ABCG2_ 421 nt C > A	rs2231142	0.00 + −0.00	0.36 + −0.01	0.00 + −0.00	0.00 + −0.00	0.01 + −0.00	0.00 + −0.00		0.35 + −0.01	0.00 + −0.00	0.00 + −0.00	0.00 + −0.00
		Solute carrier family 15 (H+/peptide transporter)												
	8	SLC15A2_P409S	rs1143671	0.04 + −0.00	0.09 + −0.00	0.00 + −0.00	0.00 + −0.00	0.00 + −0.00	0.00 + −0.00	0.01 + −0.00	0.00 + −0.00	0.03 + −0.00	0.00 + −0.00	0.67 + −0.01
	9	SLC15A2_R509K	rs1143672		0.38 + −0.01	0.00 + −0.00			0.00 + −0.00					0.62 + −0.01
	10	SLC15A2_A284A	rs2293616	0.11 + −0.01	0.19 + −0.01	0.00 + −0.00	0.00 + −0.00	0.00 + −0.00	0.00 + −0.00	0.08 + −0.01	0.00 + −0.00	0.09 + −0.0073	0.00 + −0.00	0.57 + −0.01
	11	SLC15A2_L350F	rs2257212	0.04 + −0.00	0.09 + −0.01	0.00 + −0.00	0.00 + −0.00	0.00 + −0.00	0.00 + −0.00	0.01 + −0.00	0.00 + −0.00	0.05 + −0.00	0.00 + −0.00	0.67 + −0.01
		Solute carrier family 22 (organic cation transporter)												
	12	SLC22A1_P283L	rs4646277				0.20 + −0.00		0.55 + −0.01					
	13	SLC22A1_P341L	rs2282143	0.82 + −0.00	0.04 + −0.00	0.00 + −0.00	0.00 + −0.00	0.11 + −0.00	0.00 + −0.00	0.12 + −0.01	0.63 + −0.00	0.70 + −0.00		0.03 + −0.00
	14	SLC22A1_M408V	rs628031	0.47 + −0.01	0.17 + −0.01	0.00 + −0.00	0.26 + −0.01	0.91 + −0.00	0.00 + −0.00	0.09 + −0.00	0.00 + −0.00	0.07 + −0.00	0.61 + −0.01	0.02 + −0.00
	15	SLC22A2_K432Q	rs8177517	1.00 + −0.00	0.37 + −0.00	0.60 + −0.01			0.61 + −0.0026	0.00 + −0.00		0.76 + −0.01		0.00 + −0.00
	16	SLC22A2_M165I	rs8177507	1.00 + −0.00	0.29 + −0.00	0.47 + −0.00			1.00 + −0.00		0.67 + −0.01			0.37 + −0.01
	17	SLC22A2*4.1_S270A	rs316019	0.00 + −0.00	0.21 + −0.01	0.09 + −0.01	0.26 + −0.00	0.02 + −0.00	0.16 + −0.00	0.00 + −0.00	1.00 + −0.00	0.01 + −0.00	0.01 + −0.00	0.00 + −0.00
		Solute carrier organic anion transporter family												
	18	SLCO1B1*1B_N130D	rs2306283	0.00 + −0.00	0.00 + −0.00	0.00 + −0.00	0.00 + −0.00	0.66 + −0.01	0.21 + −0.01	0.00 + −0.00	0.00 + −0.00	0.00 + −0.00	0.00 + −0.00	0.00 + −0.00
	19	SLCO1B1*5_V174A	rs4149056	0.01 + −0.00	0.26 + −0.01	0.54 + −0.01	0.70 + −0.01	0.00 + −0.00	0.39 + −0.00	0.00 + −0.00	0.16 + −0.01	0.28370 + −0.0066	0.08 + −0.00	0.00 + −0.00
	20	SLCO1B3_699G > A	rs7311358		0.01 + −0.00	0.93 + −0.00			0.12 + −0.02					0.00 + −0.00
	21	SLCO1B3_334T > G	rs4149117	0.00 + −0.00	0.04 + −0.0020	0.28 + −0.01	0.84 + −0.00	0.00 + −0.00	0.13 + −0.01	0.00 + −0.00	0.06 + −0.0041	0.00 + −0.00	0.01 + −0.00	0.00 + −0.00
	22	SLCO2B1*3_S486F	rs2306168	0.00 + −0.00	0.00 + −0.00	0.13 + −0.00	0.06 + −0.00	0.23 + −0.01	0.00 + −0.00	0.00 + −0.00	1.00 + −0.00	0.00 + −0.00	0.00 + −0.00	0.00 + −0.00
phase II metabolizing enzymes		Glutathione S-transferase pi 1												
	23	GSTP1_V105I	rs1695	0.00 + −0.00	0.00 + −0.00	0.76 + −0.00	0.57 + −0.01	0.00 + −0.00	0.00 + −0.00	0.00 + −0.00	0.00 + −0.00	0.00 + −0.00	0.02 + −0.00	0.00 + −0.00
		N-acetyltransferase												
	24	NAT1*11A-C g.-344C > T	rs4986988		0.20 + −0.01	0.31 + −0.00	0.31 + −0.00		0.74 + −0.00	0.31 + −0.01	0.70 + −0.00	0.23 + −0.01	0.15 + −0.01	0.20 + −0.01
	25	NAT1*14_560G > A	rs4986782		0.13 + −0.01	1.00 + −0.00			1.00 + −0.00				0.55 + −0.00	1.00 + −0.00
	26	NAT2*5_341T > C	rs1801280		0.00 + −0.00	0.00 + −0.00			0.00 + −0.00					0.80 + −0.00
	27	NAT2*6_590G > A	rs1799930	0.34 + −0.01	0.14 + −0.00	0.08 + −0.01	0.98 + −0.00	0.03 + −0.00	0.35 + −0.01	0.48 + −0.01	0.29 + −0.01	0.31 + −0.01	0.48 + −0.01	0.93 + −0.00
	28	NAT2*7_857G > A	rs1799931	0.16 + −0.00	0.00 + −0.00	0.02 + −0.00	0.09 + −0.00	0.17 + −0.01	0.14 + −0.00	0.00 + −0.00	0.12 + −0.00	0.01 + −0.00	0.00 + −0.00	0.00 + −0.00
	29	NAT2*11_481C > T	rs1799929	0.89 + −0.00	0.00 + −0.00	0.00 + −0.00	0.00 + −0.00	0.13 + −0.00	0.00 + −0.00	0.04 + −0.00	0.12 + −0.01	0.00 + −0.00	0.00 + −0.00	0.02 + −0.00
	30	NAT2*12_803A > G	rs1208	0.08 + −0.01	0.00 + −0.00	0.00 + −0.00	0.00 + −0.00	0.00 + −0.00	0.00 + −0.00	0.00 + −0.00	0.00 + −0.00	0.00 + −0.00	0.00 + −0.00	0.01 + −0.00
	31	NAT2*13_282C > T	rs1041983	0.10 + −0.00	0.03 + −0.00	0.65 + −0.01	0.27 + −0.01	0.54 + −0.01	0.03 + −0.00	0.09 + −0.01	0.47 + −0.01	0.45 + −0.01	0.15 + −0.01	0.00 + −0.00
		Thiopurine S-methyltransferase												
	32	TPMT*3C_719A > G C240Y	rs1142345	0.00 + −0.00	0.36 + −0.01	0.68 + −0.00	0.31 + −0.01	0.50 + −0.00	0.73 + −0.00	0.00 + −0.00	0.00 + −0.00	0.57 + −0.00	0.69 + −0.00	0.05 + −0.00
		UDP glucuronosyltransferase												
	33	UGT1A1*6_211G > A	rs4148323		0.00 + −0.00	0.02 + −0.00	0.62 + −0.01	0.00 + −0.00	0.73 + −0.01		0.00 + −0.00			0.00 + −0.00
	34	UGT2B7*2b_-327G > A	rs7662029	0.00 + −0.00	0.43 + −0.01	0.00 + −0.00	0.00 + −0.00	0.04 + −0.01	0.00 + −0.00	0.00 + −0.00	0.00 + −0.00	0.00 + −0.00	0.34 + −0.01	0.00 + −0.00
	35	UGT1A1*60_-3263 T > G	rs4124874	0.00 + −0.00	0.35 + −0.01	0.01 + −0.00	0.30 + −0.01	0.00 + −0.00	0.18 + −0.01	0.00 + −0.00	0.05 + −0.00	0.00 + −0.00	0.87 + −0.00	0.00 + −0.00
	36	UGT2B7*2a_-161C > T	rs7668258	0.00 + −0.00	0.57 + −0.01	0.00 + −0.00	0.00 + −0.00	0.06 + −0.01	0.00 + −0.00	0.00 + −0.00	0.00 + −0.00	0.00 + −0.00	0.39 + −0.01	0.00 + −0.00
phase I metabolizing														
enzymes		Dihydropyrimidine dehydrogenase												
	37	DPYD*2A_IVS14 + 1G > A	rs3918290					0.04 + −0.00						
	38	DPYD*9A_C29R	rs1801265	0.00 + −0.00	0.86 + −0.00	0.00 + −0.00	0.00 + −0.00	0.00 + −0.00	0.00 + −0.00	0.00 + −0.00	0.02 + −0.00	0.00 + −0.00	0.02 + −0.00	0.00 + −0.00
		Cytochrome P450												
	39	CYP1A1*2 g.2455A > G	rs1048943	0.00 + −0.00	0.00 + −0.00	0.08 + −0.01	0.07 + −0.01	0.07 + −0.00	0.28 + −0.01		0.05 + −0.00		0.00 + −0.00	0.00 + −0.00
	40	CYP1A1*4 g.2453C > A	rs1799814		0.80 + −0.00	0.33 + −0.00		0.00 + −0.00	0.39 + −0.00		0.75 + −0.00			0.28 + −0.00
	41	CYP1A2*1 F g.-163C > A	rs762551	0.67 + −0.00	0.31 + −0.01	0.63 + −0.01	0.39 + −0.01	0.00 + −0.00	0.25 + −0.01	0.00 + −0.00	0.20 + −0.01	0.00 + −0.00	0.60 + −0.01	0.05 + −0.01
	42	CYP1A2*1 K g.-729C > T	rs12720461					0.40 + −0.00						
	43	CYP2A6*2 g.1799 T > A	rs1801272		0.05 + −0.00	0.60 + −0.00			0.62 + −0.00					0.37 + −0.00
	44	CYP2B6*6 g.15631G > T	rs3745274	0.94 + −0.00	0.88 + −0.00	0.03 + −0.00	0.00 + −0.00	0.00 + −0.00	0.03 + −0.00	0.41 + −0.01	0.61 + −0.00	0.01 + −0.00	0.72 + −0.00	0.00 + −0.00
	45	CYP2B6*16 g. 21011 T > C	rs28399499	0.01 + −0.00						0.00 + −0.00	0.48 + −0.01	0.16 + −0.00		0.00 + −0.00
	46	CYP2C8*2 g.11054A > T	rs11572103		1.00 + −0.00	1.00 + −0.00			1.00 + −0.00					0.00 + −0.00
	47	CYP2C8*3 g.30411A > G	rs10509681	1.00 + −0.00	0.00 + −0.00	0.04 + −0.00		0.22 + −0.01	0.03 + −0.00		0.04 + −0.00	0.12 + −0.01	0.00 + −0.00	0.01 + −0.00
	48	CYP2C8*4 g.11041C > G	rs1058930					0.69 + −0.00						
	49	CYP2C9*2 g.3608C > T	rs1799853		0.03 + −0.00	0.02 + −0.00			0.02 + −0.00					0.01 + −0.00
	50	CYP2C9*3 g.42614A > C	rs1057910	0.08 + −0.01	0.42 + −0.01	0.37 + −0.01	0.39 + −0.01	0.02 + −0.00	0.02 + −0.00		0.51 + −0.00		0.72 + −0.01	0.00 + −0.00
	51	CYP2C9*12 g.50338C > T	rs9332239		0.01 + −0.00	0.02 + −0.00			0.02 + −0.00					0.01 + −0.00
	52	CYP2C19*2 g.19154G > A (splicing defect)	rs4244285		0.10 + −0.01	0.03 + −0.00			0.03 + −0.00					0.29 + −0.01
	53	CYP2C19*2 g.80160C > T	rs3758580		0.40 + −0.01	0.01 + −0.00			0.05 + −0.00					0.58 + −0.00
	54	CYP2C9*11 g.42542C > T	rs28371685					0.42 + −0.00						
	55	CYP2C19*17 g.-806C > T	rs12248560		0.00 + −0.00	0.04 + −0.00			0.00 + −0.00					0.00 + −0.00
drug targets		Vitamin K epoxide reductase complex												
	56	VKORC1	rs8050894		0.00 + −0.00	0.00 + −0.00			0.00 + −0.00					0.00 + −0.00

Significance level = 0.05

Next, we performed a comparative analysis of the differences in genotype frequencies among the Kazakh population and data for world populations collected from the HapMap database. For individuals of African ancestry living in the southwest USA (ASW), only 35 SNPs of a total of 56 were analysed. The remaining data for this population were no included in the HapMap database. Twenty of these 35 SNPs were significantly different from those in the Kazakh population. These genes encoded drug transporters (*ABCB1*, *ABCC2*, *ABCG2*, *SLC15A2*, *SLC22A2*, *SLCO1B1*, *SLCO1B3*, and *SLCO2B1*) and phase I (*DPYD*, *CYP1A1*, and *CYP2B6*) and II (*GSTP1*, *TPMT*, *UGT2B7*, and *UGT1A1*) drug metabolic enzymes. However, we found that there were no significant differences in SNPs within genes belonging to the acetyltransferase family (*NAT2*).

For Utah residents with Northern and Western European ancestry from the CEPH collection (CEU), population analysis was carried out for 50 SNPs; 21 of these SNPs showed significant differences compared with the Kazakh population. These SNPs were found in genes encoding drug transporters (*ABCB1*, *ABCC2*, *SLC22A1*, *SLCO1B1*, *SLCO1B3*, and *SLCO2B1*) and phase I (*CYP1A1*, *CYP2C8*, *CYP2C9*, and *CYP2C19* ) and II (*NAT2*, *GSTP1*, and *UGT1A1*) drug metabolic enzymes. SNPs in genes belonging to the solute carrier family 15 (H+/peptide transporter) did not show differences between the Kazakh and CEU populations.

Only 26 of 50 SNPs showed significant differences among the Kazakh population and the Han Chinese population in Beijing, China (CHB). For the Chinese population in Metropolitan Denver, CO (CHD), population analysis was carried out for 34 SNPs; 14 of these SNPs showed significant differences from the Kazakh population. Significant differences were also observed for 24 of 51 SNPs in the Japanese population in Tokyo, Japan (JPT), 23 of 30 SNP in the Luhya population in Webuye, Kenya (LWK), 14 of 37 SNPs for the population of Mexicans in Los Angeles, CA (MEX), 21 of 33 SNPs for the population of Maasai in Kinayawa, Kenya (МKK), 17 of 33 SNPs the Tuscan population in Italy (TSI), and 36 of 50 SNPs in the Yoruban population in Ibadan, Nigeria (YRI).

For the Gujarati Indian population in Houston, TX (GIH), population analysis was carried out for 38 SNPs; 23 of these SNPs showed significant differences from the Kazakh population. Notably, comparative analyses of rs12720461, rs28371685, rs1058930, and rs3918290 were carried out only for the GIH population because frequency data in the HapMap database were only available for this population. Of these SNPs, only rs3918290 showed a significant difference from the Kazakh population.

If we compare the ratios of significantly different SNPs with the amount of data (i.e., the number of SNPs that were analysed) for each population, the YRI population showed the greatest differences compared with the Kazakh population. However, similar to the CEU population, statistically significant differences for SNPs of genes belonging to the solute carrier family 15 (H+/peptide transporter) were not found.

The SNPs rs8177507, rs3740066, rs4986988, rs4986782, rs12720461, rs1799930, rs28371685, rs4646277, rs1801272, rs11572103, and rs1058930 showed no significant differences with any of the compared populations, suggesting that the power of the study (320 DNA samples) may be insufficient.

### Linkage disequilibrium (LD) analysis for the Kazakh population

**Fig. 1:**
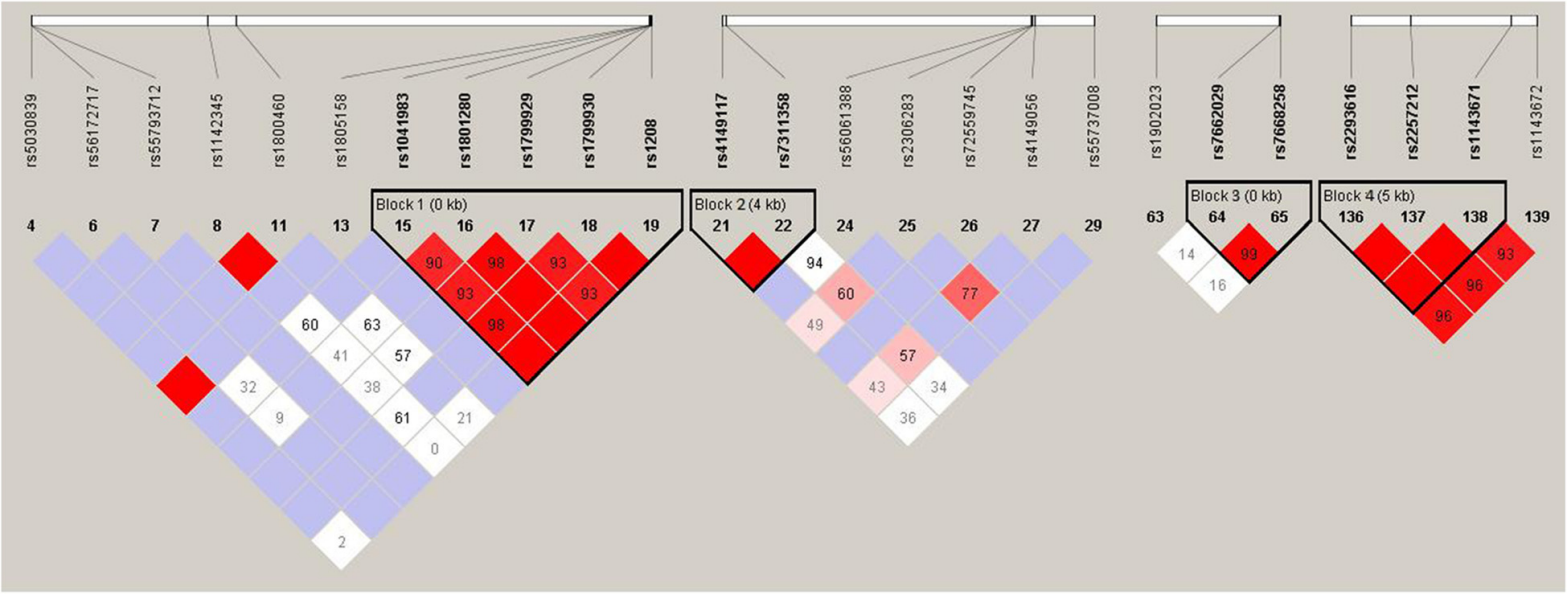
LD SNP plot. The LD is displayed according to standard colour schemes, with bright red for very strong LD (LOD > 2, D’ = 1), light red (LOD > 2, D’ < 1) and blue (LOD < 2, D’ = 1) for intermediate LD, and white (LOD < 2, D’ < 1) for no LD

Using Haploview 4.2 software, LD statistics results for the Kazakh population were obtained (Fig. 1). For block generations, the Confidence Intervals default algorithm was used. We selected SNPs that were consistent with Hardy-Weinberg equilibrium and ignored those with minor allele frequencies (MAFs) of less than 0.05. As a result, four haplotype blocks were defined: two blocks consisting of two SNPs, i.e., rs7662029 and rs7668258 (block 3, chromosome 4) and rs4149117 and rs7311358 (block 2, chromosome 12); one block consisting of three SNPs, i.e., rs2293616, rs2257212, and rs1143671 (block 4, chromosome 3); and one block consisting of five SNPs, i.e., rs1041983, rs1801280, rs1799929, rs1799930, and rs1208 (block 1, chromosome 8). The strongest LDs were found for rs2293616–rs2257212, rs2293616–rs1143671, rs2257212–rs1143671, rs7662029–rs7668258, rs4986893–rs17886522, and rs10509681–rs11572080 in the Kazakh population. The haplotype frequencies in the studied population are presented in Table 3.

**Table 3:** Haplotype frequencies in the Kazakh population

Locus	Haplotype	Frequencies
Block 1 rs1041983|rs1801280|rs1799929|rs1799930|rs1208 NAT2*13/ NAT2*5/ NAT2*11/ NAT2*6/ NAT2*12	CCTGG	0.236
	CCCGG	0.012
	TTCAA	0.248
	TTCGA	0.113
	CTCGA	0.363
	CCTGA	0.016
Block2 rs4149117|rs7311358 SLCO1B3_334T > G/ SLCO1B3_699G > A	TG	0.213
	GG	0.030
	GA	0.758
Block 3 rs7662029|rs7668258 UGT2B7*2b/ UGT2B7*2a	GC	0.464
	AT	0.525
Block 4 rs2293616|rs2257212|rs1143671 SLC15A2_A284A/ SLC15A2_L350F/ SLC15A2_P409S	GCC	0.449
	ATT	0.546

The crossover percentage matrix showed that the highest value had the pattern GA-AT (block 2–block 3; 40.4 %). Additionally, 34.5 % of all samples had the pattern GA-GC (block 2–block 3), 28.5 % had the pattern AT-ATT GC (block 3–block 4), and 26.9 % had the pattern CTCGA-GA (block 1–block 2).

Tag-SNP analysis was also carried out using the aggressive tagging strategy (r^2^ threshold: 0.8, logarithm (base 10) of odds [LOD] threshold: 3.0, minimum distance between tags: 0 kb). The analysis results are shown in Table 4. We found that rs1143672 was a tag-SNP for block 4. Therefore, it was likely that block 4 was formed by four SNPs, i.e., rs2293616, rs2257212, rs1143671, and rs1143672, rather than three SNPs.

**Table 4:** Tag SNPs

#	Test	Alleles Captured	Chromosome
1	rs1143672	rs2257212. rs2293616. rs1143671. rs1143672	3
2	rs72558190	rs72558190. rs41291556. rs28371686	10
3	hCV32407240	rs72549346. rs5030655. hCV32407240	22
4	rs1208	rs1801280. rs1208. rs1799929	8
5	rs4986893	rs17886522. rs4986893	10
6	rs4149117	rs4149117. rs7311358	12
7	rs9332239	rs9332239. hCV72649992	10
8	rs1805158	rs1805158. rs5030839	8
9	rs10509681	rs10509681. rs11572080	10
10	rs1058930	rs1058930. rs11572103	10
11	rs3758580	rs4244285. rs3758580	10
12	rs41279854	rs10264272. rs41279854	7
13	rs55640102	rs55640102. rs9332131	10
14	rs8177507	rs55918055. rs8177507	6
15	rs7668258	rs7662029. rs7668258	4

### Comparative analysis of haplotype frequency

**Fig. 2:**
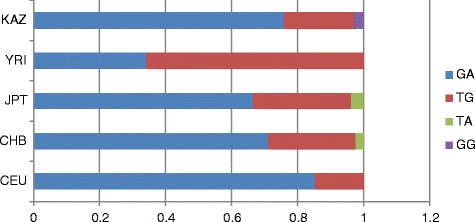
Haplotype analysis results of rs4149117 and rs7311358 in the *SLCO1B* gene (chromosome 12)

Next, we carried out a comparative analysis of the haplotype frequencies of the samples from the Kazakh population and published data from the HapMap database, including 11 worldwide populations. All of the SNPs described in Fig. 1 were used for analysis; however, not all of these SNPs were present in the HapMap database. For block generations, the Confidence Intervals default algorithm was used (Haploview 4.2, MAF < 0.05). Block generation results for all 11 population are presented in Additional file 3. From these data, only the CEU population formed a block in the *NAT2* gene that was similar to that in the Kazakh population, consisting of rs1041983, rs1801280, rs1799929, rs1799930, and rs1208. The CEU block contained seven haplotypes, whereas that in the Kazakh population contained only six haplotypes; additionally, the frequencies were different (Table 5). The GIH, LWK, MKK, and TSI populations generated blocks consisting of only four SNPs: rs1041983, rs1799929, rs1799930, and rs1208, whereas the MEX and YRI populations generated blocks consisting of three SNPs (rs1041983, rs1799929, and rs1799930). The JPT population generated blocks consisting of two SNPs (rs1041983 and rs1799930). Blocks were not generated by ASW, CHB, or CHD populations. Additionally, CEU, CHB, JPT, and YRI populations generated blocks similar to those of the Kazakh population, consisting of two SNPs (rs4149117 and rs7311358) in the *SLCO1B3* gene (Additional file 3). These populations had four haplotypes that differed in frequency (Fig. 2). The highest frequency of haplotype GA was found in the CEU population (0.852), whereas the lowest frequency of haplotype GA was found in the YRI population (0.342). The value closest to that in the Kazakh population for haplotype GA (0.758) was found in the CHB population (0.710). The highest and lowest frequencies of haplotype TG were found in the YRI (0.658) and CEU (0.148) populations. The value closest to the Kazakh population for haplotype TG (0.213) was found in the CHB population (0.265). The TA haplotype was found only in the JPT (0.038) and CHB (0.025) populations, and the GG haplotype was found only in the Kazakh population (0.030). The rest of the analysed populations did not generate blocks.

**Table 5:** Haplotype analysis results of rs1041983, rs1801280, rs1799929, rs1799930 and rs1280 in *NAT2* (chromosome 8)

Population	CEU	GIH	JPT	LWK	MEX	MKK	TSI	YRI	KAZ
CCTGG	0.392								0.236
TTCAA	0.294								0.248
CTCGA	0.206								0.363
CCTGA	0.040								0.016
CCCGG	0.029								0.012
CTCGG	0.020								
TTCGA	0.020								0.113
CCGA		0.205		0.086		0.075	0.233		
CCAA				0.014					
TCAA		0.352		0.279		0.301	0.284		
CTGG		0.322		0.322		0.451	0.443		
TCGA		0.062		0.159		0.112	0.017		
CCGG		0.059		0.139		0.061	0.011		
CG			0.679						
TA			0.238						
TG			0.083						
CTG					0.375				
CCG					0.320				
TCA					0.185				
TCG					0.120				
TTC								0.478	
CTC								0.239	
CCT								0.186	
CCC								0.097	

**Fig. 3:**
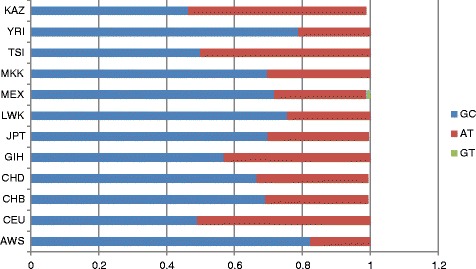
Haplotype analysis results of rs7662029 and rs7668258 in the *UGT2* gene (chromosome 4)

Kazakh population block, consisting of rs7662029 and rs7668258 in the *UGT2B7* gene, was found in all 11 populations (Additional file 3). The highest and lowest frequencies of haplotype GC were found in the YRI (0.824) and CEU (0.490) populations, and the highest and lowest frequencies of haplotype AT were found in the CEU (0.510) and AWS (0.176) populations, respectively. The GC (0.464) and AT (0.525) haplotype frequencies in the Kazakh population were close to the respective frequencies in the CEU population (Fig. 3).

**Fig. 4:**
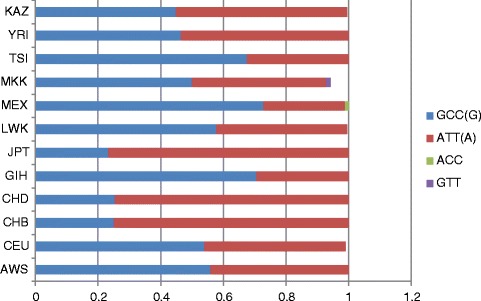
Haplotype analysis results of rs2293616, rs2257212, and rs1143671 in the *SLC15A2* gene (chromosome 3)

All 11 populations generated blocks in the *SLC15A2* gene (Additional file 3). However, these blocks contained different numbers of SNPs. The CEU, CHB, JPT, and YRI populations generated blocks consisting of four SNPs: rs2293616, rs2257212, rs1143671, and rs1143672. The blocks of the other analysed populations consisted of three SNPs: rs2293616, rs2257212, and rs1143671. The highest and lowest frequencies of haplotype GCC were found in the MEX (0.728) and CEU (0.253) populations (Fig. 4). The highest and lowest frequencies of haplotype GCCG were found in the CEU (0.540) and JPT (0.233) populations. The highest frequencies of haplotypes ATT and ATTA were found in the CHD (0.747) and CHB (0.750) populations, whereas the lowest frequencies of haplotypes ATT and ATTA were found in the GIH (0.295) and CEU (0.450) populations.

If we take into account rs1143672 tagging analysis results of the Kazakh population and assume that block 4 consisted of four SNPs, the frequency of the GCCG haplotype was 0.459, and that of ATTA was 0.537. These values were nearly identical to the results of the YRI population.

## Discussion

In this study, we examined the frequencies of specific SNPs in the Kazakh population and compared the results with those in the HapMap database for 11 other populations throughout the world. The results showed a fairly high percentage of population differentiation, providing insights into the different racial groups that may have contributed to the Kazakh population.

The Kazakh population is an interesting model in population genetics, and the process through which the Kazakh population formed is poorly understood. However, some scientists believe that the Kazakh population was formed by the mixing of the Asian and Caucasoid populations [6] owing to the observation that there are Kazakh individuals who have distinctive Asian and/or Caucasoid traits. Additionally, the Kazakh people are divided into three Zhuzes and further divided into distinct tribes in each Zhuz. The historical division into Zhuzes could be argued on the basis of the different origins of each Zhuz; this could explain the different frequencies of SNPs within the population. However, in our previous study, in which we had a larger sample collection, we compared the frequencies of SNPs within the three Zhuzes and found no significant differences in SNPs between Zhuzes [7]. Thus, we concluded that we could combine all samples in one sample collection.

Genotyping of 158 SNPs from 320 DNA samples showed that 75 SNPs were not found in the studied samples (Table 1, Additional file 2). The frequencies of many of these SNPs were very low in other populations as well [10]. However, we could not conclude that these SNPs did not occur (or were only present in a very low frequency) in the Kazakh population. In addition, seven of 83 SNPs identified in the Kazakh population were not in Hardy-Weinberg equilibrium. We expect that this result may have been caused by the insufficient power of the study.

In this study, we selected SNPs involved in the ADME of drugs for genotyping. Thus, 19 of 83 SNPs occurring in the Kazakh population were associated with drugs used in the treatment of cardiovascular diseases (statins, beta-blockers, anticoagulants, and antiplatelet agents). The recommended dosage for the cholesterol-lowering agent simvastin is 80 mg (U.S. Food and Drug Administration [FDA], www.fda.gov). Moreover, the FDA recommends dose correction when using simvastatin with certain drugs that cause increased concentrations of simvastatin, resulting in increased risk of myopathy. In patients with the C allele at the SNP rs4149056 in the *SLCO1B1* gene, there are modest increases in myopathy risk even at lower doses of simvastatin (40 mg daily); if optimal efficacy is not achieved with a lower dose, alternate agents should be considered [11]. The TT genotype frequency in our study was 72 % in Kazakhs, compared with 91 %, 71 %, 60 %, and 98 % in the ASW, CHB, TSI, and YRI populations, respectively. Moreover, responses of individuals to statin drugs are associated with *ABCB1* (rs2032582), *ABCC2* (rs717620), *ABCG2* (rs2231142), *SLCO1B1* (rs2306283), *CYP2C8* (rs10509681), and *CYP2C9* (rs1799853, rs1057910). Comparative analysis of the frequencies of these SNPs in the Kazakh population with those in the ASW population showed significant differences for all SNPs, except for the SNPs in cytochrome P450. In contrast, for the CEU population, only the SNPs in cytochrome P450 and *SLCO1B1* (rs2306283) were significantly different from those in the Kazakh population.

The *VKORC1* gene on chromosome 16 is one of the main genes associated with the dosage of coumarin anticoagulants, and several mutations in this gene are associated with enzyme deficiency. An allelic variant in *VKORC1* (c.-1639G > A) determines up to 30 % of the variability in warfarin dosage [12, 13]. In a previous study, the *VKORC1* c.-1639G > A mutation was found to be linked with *VKORC1* c. 173 + 1369G > C (rs8050894) and *VKORC1* c. 173 + 1000C > T (rs9934438) mutations [14]. Subjects carrying the 1173 T (rs9934438) allele required a lower maintenance dose of warfarin compared with that in subjects harbouring the CC genotype in African Americans and Caucasians. Before reaching the maintenance dose, only Caucasians with the T allele had a significantly increased risk of international normalized ratio compared with that in Caucasians harbouring the CC genotype. Polymorphisms in the *VKORC1* gene are associated with the maintenance dose requirements of warfarin among both African Americans and Caucasians [15]. Interestingly, in *VKORC1*, the allele frequency of rs8050894 c. 173 + 1369G > C is as high as 94 % (G allele) in Asian populations, whereas that in Caucasians is about 37 % (G allele). In the Kazakh population, we found that the frequency of allele G was 63 %. Importantly, the response to anticoagulant drugs (e.g., warfarin) is associated with *CYP1A1* (rs1048943) and *CYP2C9* (rs1057910, rs28371685, and rs1799853). Comparative analysis of the frequencies of these SNPs showed that all of the SNPs listed above were significantly different between the Kazakh population and the YRI population, with the exception of rs28371685. The majority of the data were not present in the HapMap database (Table 2).

The treatment of cardiovascular diseases often involves administration of Plavix (clopidogrel). The influence of genetics on the pharmacokinetic and pharmacodynamic response to clopidogrel has been examined in previous studies [16]. Several polymorphic P450 enzymes are involved in the activation of clopidogrel. The CYP2C19 isoenzyme is involved in the formation of an active metabolite and intermediate metabolite, 2-oxoclopidogrel. The pharmacokinetics and antiplatelet effects of the active metabolite of clopidogrel, which were investigated by means of platelet aggregation *ex vivo*, vary depending on the genotype of the CYP2C19 isoenzyme. Allele CYP2C19*1 is responsible for the normally functioning metabolism, whereas alleles of the CYP2C19*2 and CYP2C19*3 genes are responsible for decreased metabolism. The frequency of the A (rs4244285) allele in our study was 17 % in Kazakhs, compared with 15.5 %, 28 %, and 14 % in CEU, JPT, and YRI populations, respectively. For rs4986893, the A allele frequency in our study was 4 % in Kazakhs; no HapMap data were available for other populations. Other alleles associated with reduced metabolism have been identified in CYP2C19*4, CYP2C19*5, CYP2C19*6, CYP2C19*7, and CYP2C19*8; however, these alleles were rarely found in our population.

The response to antiplatelet agents (Plavix) is also associated with *ABCB1* (rs2032582), *CYP1A1* (rs1048943), *CYP1A2* (rs762551), *CYP2B6* (rs3745274), *CYP2C8* (rs10509681), *CYP2C9* (rs1799853), and *CYP2C19* (rs12248560). Comparative analysis of SNP frequencies showed that these SNPs were significantly different between the Kazakh population and the YRI population, with the exception of rs2032582. The majority of data were not available in the HapMap database. Significant differences in genes in the ATP-binding cassette system were not found between the Kazakh and JPT populations (Table 2).

Labetalol is a nonselective β-adrenergic antagonist with additional α1-adrenergic antagonist properties. *CYP2C19* is involved in the metabolism of several important groups of drugs, including a number of β-blockers, such as propranolol and labetalol [17]. A previous study showed that the activity of labetalol is significantly affected by common *CYP2C19* polymorphisms in individuals of Chinese ethnicity; specifically, subjects with the CYP2C19*2/*2 (rs4244285) genotype had a higher peak and area under the concentration-time curve than subjects with the CYP2C19*1/*1 genotype, and heterozygotes had intermediate values [18]. In the Kazakh population, genotype AA was found in 2 % of individual, whereas 5.2 %, 6.8 %, and 3.4 % of individuals in the CEU, JPT, and YRI populations carried this allele.

Responses to β-blockers are associated with *ABCB1* (rs1128503) and *UGT1A1* (rs4148323 and rs4124874). All of these SNPs were significantly different between the Kazakh and YRI populations, although most data were not available in the HapMap database. Significant differences in genes in the ATP-binding cassette system and UDP glucuronosyltransferase were not observed between the Kazakh and JPT populations. Moreover, SNPs in the *UGT1A1* genes did not differ between the CHD and TSI populations (Table 2).

SNPs in *ABCB1* (rs1045642) and *CYP2C19* (rs4244285) are associated with the response to β-blockers, anticoagulants, and antiplatelet agents. Importantly, the frequencies of these SNPs were significantly different between the Kazakh population and the ASW, CEU, GIH, MKK, and YRI populations for rs1045642 and between the Kazakh population and the CHB and JPT populations for rs4244285 (Table 2).

Analysis of the results of haplotype frequencies among the populations examined in this study showed substantial and significant variations. For example, only four populations generated the block in the *SLCO1B3* gene, similar to the Kazakh population. The CHB population had the most similar haplotype frequency compared with the Kazakh population. However, there were variations in haplotypes among populations, with differences in GA, TG, and TA haplotypes for the CHB and in GA, TG, and GG haplotypes in the Kazakh population. Only eight populations generated blocks in the *NAT2* gene, and 24 haplotypes were formed by the analysed SNPs. From these results, none of the examined populations were similar to the Kazakh population with regard to this gene. However, all 11 populations generated haplotype blocks in *UGT2B7* and *SLC15A2* genes, and the CEU population had the closest frequency for *UGT2B7*, whereas the YRI population had the closest frequency for *SLC15A2* relative to the Kazakh population. Thus, for these three genes (*UGT2B7*, *SLC15A2*, and *SLCO1B3*), the Kazakh population showed similarities with three different populations. All three of these populations showed significant differences in these three genes.

## Conclusion

In summary, our data provided important information for personalised medicine in the Kazakh population, supporting the genotyping of specific SNPs before administration of drugs with respect to the patient’s ethnicity. The allele frequencies of the studied SNPs were quite different in the Kazakh population compared with those for all of the other populations examined. Moreover, we could not classify the Kazakh population as Asian or Caucasian, indicating that the Kazakh population may have been formed from several populations belonging to different racial groups.

Our study had several limitations. First, we had only a small number of samples. In addition, it will be useful to perform comparative analysis of the frequencies of SNPs in the different Zhuzes in order to clarify that combining samples from all Zhuzes is acceptable. Unfortunately, in this study, we did not have sufficient data to classify individuals into Zhuzes, only by nationality. In future studies, we plan to increase the number of samples and to examine additional SNPs.

## Methods

### Characteristics of the study populations

A total of 320 individuals living in Astana during 2012–2013 and belonging to the Kazakh nationality participated in this study. All individuals included in the present study were unrelated and randomly selected from different regions of Kazakhstan. The mean (± standard deviation [SD]) age of the participants was 44.06 ± 17.98 years (age range: 19–86), and the population included 239 men and 81 women.

Blood samples were collected in clinics in the city of Astana (Republican research center of transfusion, National research cardiac surgery center and Medical center of the Presidential Administration of Kazakhstan). Blood samples were taken according to the study protocol, which was approved by the Ethics Committee of the National Center for Biotechnology of the Republic of Kazakhstan, Astana, Kazakhstan (No. 11, 14.02.2010), Republican research center of transfusion, National research cardiac surgery center and Medical center of the Presidential Administration of Kazakhstan.

Each participant was informed of the purpose and methods of the study, and written informed consent was obtained from all participants. Each volunteer filled out a questionnaire to collect standard personal data, including their nationality and the nationalities of their parents and grandparents. Based on the concept of Zhety ata, in which each Kazakh individual is expected to know seven generations of their ancestors, we were able to collect information on nearly seven generations from each volunteer. While the questionnaire included data only to the second generation, the ethnicities of ancestors from the third to seventh generations were determined according to a verbal survey. If an individual indicated that he or she had an ancestor who was not a Kazakh, the blood sample from this individual was excluded.

### Genotyping

DNA was collected from whole venous blood samples collected in EDTA-containing tubes. DNA from blood was extracted by the salting-out method [19], and genotyping was performed using real-time polymerase chain reaction (PCR) with high-throughput OpenArray technology. Amplification was performed on a QuantStudio 12 K Flex thermocycler (Life Technologies, USA) using pharmacogenomic PGx panels. The composition of the PCR mixture was as follows: OpenArray Genotyping Master Mix (2.5 μL/sample) and DNA sample of 50 ng/μL (2.5 μL/sample). The reaction volume was 5 μL. Each reaction mixture was covered by immersion oil. The PCR conditions were as follows: 10 min at 93°С; 50 cycles of 45 s at 93°С, 13 s at 94°С, and 2.14 min at 53.5°С; and incubation at 25°С for 2 min. Data processing was carried out using TaqMan Genotyper Software v. 1.3.

### Statistical analysis

Statistical analysis was performed using Haploview 4.2 [20] and Arlequin 3.1 [21] software. The correspondence of the distributions of genotype frequencies to the Hardy-Weinberg equilibrium was assessed using the χ^2^ criterion (preliminary analysis) and exact tests using a Markov chain. Data from the HapMap database were used for the comparative analysis of the differences in genotype and haplotype frequencies among Kazakh and world populations (HapMap Genome Browser release #27 [Phases 1, 2, & 3 - merged genotypes and frequencies]) [10]. The exact test of population differentiation (Markov chain) method was used for the analysis [9, 21].

## Availability of supporting data

The data sets supporting the results of this article are included within the article and its additional files.

## Additional files

Additional file 1:
**Characteristics of studied allele variants of genes.** (DOC 281 kb) Additional file 2:
**A list of SNPs that were not found in heterozygous or homozygous variants.** (DOC 83 kb) Additional file 3:
**LD SNP plot.** LD analysis of the *SLC15A2*, *UGT2B7*, *NAT2*, and *SLCO1B3* genes in 11 populations (HapMap data) and the Kazakh population (our data). A. *SLC15A2*, B. *UGT2B7*, C. *NAT2*, D. *SLCO1B3*. (DOC 2457 kb)

## Abbreviations

ADME: Absorption, distribution, metabolism, and excretion
ASW: African ancestry living in the southwest USA
CEU: Utah residents with Northern and Western European ancestry from the CEPH collection
CHB: Han Chinese population in Beijing, China
CHD: Chinese population in Metropolitan Denver, CO
GIH: Gujarati Indian population in Houston, TX
JPT: Japanese population in Tokyo, Japan
LD: Linkage disequilibrium
LWK: Luhya population in Webuye, Kenya
MAFs: Minor allele frequencies
MEX: Mexicans in Los Angeles, CA
МKK: Maasai in Kinayawa, Kenya
PCR: Polymerase chain reaction
SNPs: Single nucleotide polymorphisms
TSI: Tuscan population in Italy
YRI: Yoruban population in Ibadan, Nigeria

## References

[1] Wang SM, Zhu AP, Li D, Wang Z, Zhang P, Zhang GL (2009). Frequencies of genotypes and alleles of the functional SNPs in CYP2C19 and CYP2E1 in mainland Chinese Kazakh, Uygur and Han populations. J Hum Genet..

[2] Magalon H, Patin E, Austerlitz F, Hegay T, Aldashev A, Quintana-Murci L (2008). Population genetic diversity of the NAT2 gene supports a role of acetylation in human adaptation to farming in Central Asia. Eur J Hum Genet..

[3] Tarlykov PV, Zholdybayeva EV, Akilzhanova AR, Nurkina ZM, Sabitov ZM, Rakhypbekov TK (2013). Mitochondrial and Y-chromosomal profile of the Kazakh population from East Kazakhstan. Croat Med J..

[4] New restrictions, contraindications, and dose limitations for Zocor (simvastatin) to reduce the risk of muscle injury, 2011. U.S. Food and Drug Administration.

[5] Ministry of National Economy of the Republic of Kazakhstan. Committee on Statistics. Available at: http://www.stat.gov.kz/ (2015). Accessed 19 June 2015.

[6] Iskakova MK (2012). Kazakh’s tamga (Tamga kazakhov).

[7] Iskakova AN, Romanova AA, Voronina EN, Sikhayeva NS, Belozerceva AB, Filipenko ML (2014). Allele frequency and genotype distribution of 9 SNPs in the Kazakh population. J Pharmacogenomics Pharmacoproteomics.

[8] Guo SW, Thompson EA (1992). Performing the exact test of Hardy-Weinberg proportion for multiple alleles. Biometrics.

[9] Raymond M, Rousset F (1995). An exact test for population differentiation. Evol Bioinform Online.

[10] Thorisson GA, Smith AV, Krishnan L, Stein LD (2005). The International HapMap Project Web site. Genome Res..

[11] Wilke RA, Ramsey LB, Johnson SG, Maxwell WD, McLeod HL, Voora D (2012). The clinical pharmacogenomics implementation consortium: CPIC guideline for SLCO1B1 and simvastatin-induced myopathy. Clin Pharmacol Ther..

[12] Limdi NA, McGwin G, Goldstein JA, Beasley TM, Arnett DK, Adler BK (2008). Influence of CYP2C9 and VKORC1 1173C/T genotype on the risk of hemorrhagic complications in African-American and European-American patients on warfarin. Clin Pharmacol..

[13] Wang TL, Li HL, Tjong WY, Chen QS, Wu GS, Zhu HT (2008). Genetic factors contribute to patient-specific warfarin dose for Han Chinese. Clin Chim Acta..

[14] Rieder MJ, Reiner AP, Gage BF, Nickerson DA, Eby CS, McLeod HL (2005). Effect of VKORC1 haplotypes on transcriptional regulation and warfarin dose. N Engl J Med..

[15] Schelleman H, Chen Z, Kealey C, Whitehead AS, Christie J, Price M (2007). Warfarin response and vitamin K epoxide reductase complex 1 in African Americans and Caucasians. Clin Pharmacol Ther..

[16] Mega JL, Close SL, Wiviott SD, Shen L, Hockett RD, Brandt JT (2009). Cytochrome P-450 polymorphisms and response to clopidogrel. N Engl J Med..

[17] Höcht C, Bertera FM, Mayer MA, Taira CA (2010). Issues in drug metabolism of major antihypertensive drugs: beta-blockers, calcium channel antagonists and angiotensin receptor blockers. Expert Opin Drug Metab Toxicol..

[18] Chan SW, Hu M, Ko SS, Tam CW, Fok BS, Yin OQ (2013). CYP2C19 genotype has a major influence on labetalol pharmacokinetics in healthy male Chinese subjects. Eur J Clin Pharmacol..

[19] Miller SA, Dykes DD, Polesky HF (1988). A simple salting out procedure for extracting DNA from human nucleated cells. Nucleic Acids Res..

[20] Barrett JC, Fry B, Maller J, Daly MJ (2005). Haploview: analysis and visualization of LD and haplotype maps. Bioinformatics.

[21] Excoffier L, Laval G, Schneider S. Arlequin ver. 3.0: an integrated software package PMC2658868 19325852

