# Supplementary material for: Polymorphisms in genes involved in the absorption, distribution, metabolism, and excretion of drugs in the Kazakhs of Kazakhstan
Source: BMC Genet. 2016 Jan 19;17:23. doi: 10.1186/s12863-016-0329-x (PMC4717528; doi:10.1186/s12863-016-0329-x)
Supplement: Additional file 1: — Characteristics of studied allele variants of genes. (DOC 281 kb) [file 12863_2016_329_MOESM1_ESM.doc]

|  |
| --- |

**Additional file 1.** Characteristics of studied allele variants of genes.

| # | Gene Symbol | Gene Name | Chromosome | NCBI SNP Reference | Cytogenetic Band | SNP Type | Location on NCBI Assembly |
| --- | --- | --- | --- | --- | --- | --- | --- |
| 1 | ABCB1 | ATP-binding cassette; sub-family B (MDR/TAP); member 1 | 7 | rs1045642 | 7q21.12a | INTERGENIC/UNKNOWN | 87138645 |
| 2 | ABCB1 | ATP-binding cassette; sub-family B (MDR/TAP); member 1 | 7 | rs1128503 | 7q21.12a | SILENT MUTATION | 87179601 |
| 3 | ABCB1 | ATP-binding cassette; sub-family B (MDR/TAP); member 1 | 7 | rs2032582 | 7q21.12a | INTERGENIC/UNKNOWN | 87160618 |
| 4 | ABCB1 | ATP-binding cassette; sub-family B (MDR/TAP); member 1 | 7 | rs2032582 | 7q21.12a | INTERGENIC/UNKNOWN | 87160618 |
| 5 | ABCC2 | ATP-binding cassette; sub-family C (CFTR/MRP); member 2 | 10 | rs56296335 | 10q24.2c | MIS-SENSE MUTATION | 101610393 |
| 6 | ABCC2 | ATP-binding cassette; sub-family C (CFTR/MRP); member 2 | 10 | rs56220353 | 10q24.2c | INTERGENIC/UNKNOWN | 101578641 |
| 7 | ABCC2 | ATP-binding cassette; sub-family C (CFTR/MRP); member 2 | 10 | rs3740066 | 10q24.2c | SILENT MUTATION | 101604207 |
| 8 | ABCC2 | ATP-binding cassette; sub-family C (CFTR/MRP); member 2 | 10 | rs56199535 | 10q24.2c | INTERGENIC/UNKNOWN | 101578577 |
| 9 | ABCC2 | ATP-binding cassette; sub-family C (CFTR/MRP); member 2 | 10 | rs717620 | 10q24.2c | UTR 5 | 101542578 |
| 10 | ABCC2 | ATP-binding cassette; sub-family C (CFTR/MRP); member 2 | 10 | rs2273697 | 10q24.2c | MIS-SENSE MUTATION | 101563815 |
| 11 | ABCG2 | ATP-binding cassette; sub-family G (WHITE); member 2 | 4 | rs2231142 | 4q22.1b | MIS-SENSE MUTATION | 89052323 |
| 12 | ABCG2 | ATP-binding cassette; sub-family G (WHITE); member 2 | 4 | rs72552713 | 4q22.1b | NONSENSE MUTATION | 89052957 |
| 13 | CYP1A1 | cytochrome P450; family 1; subfamily A; polypeptide 1 | 15 | rs41279188 | 15q24.1b | MIS-SENSE MUTATION | 75012979 |
| 14 | CYP1A1 | cytochrome P450; family 1; subfamily A; polypeptide 1 | 15 | rs72547509 | 15q24.1b | MIS-SENSE MUTATION | 75013026 |
| 15 | CYP1A1 | cytochrome P450; family 1; subfamily A; polypeptide 1 | 15 | rs56313657 | 15q24.1b | INTERGENIC/UNKNOWN | 75013804 |
| 16 | CYP1A1 | cytochrome P450; family 1; subfamily A; polypeptide 1 | 15 | rs41279188 | 15q24.1b | MIS-SENSE MUTATION | 75012979 |
| 17 | CYP1A1 | cytochrome P450; family 1; subfamily A; polypeptide 1 | 15 | rs72547510 | 15q24.1b | INSERTION/DELETION | 75013093 |
| 18 | CYP1A1 | cytochrome P450; family 1; subfamily A; polypeptide 1 | 15 | rs1048943 | 15q24.1b | INTERGENIC/UNKNOWN | 75012985 |
| 19 | CYP1A1 | cytochrome P450; family 1; subfamily A; polypeptide 1 | 15 | rs1800031 | 15q24.1b | UTR 3 | 75012235 |
| 20 | CYP1A1 | cytochrome P450; family 1; subfamily A; polypeptide 1 | 15 | rs1799814 | 15q24.1b | MIS-SENSE MUTATION | 75012987 |
| 21 | CYP1A2 | cytochrome P450; family 1; subfamily A; polypeptide 2 | 15 | rs56107638 | 15q24.1b | INTERGENIC/UNKNOWN | 75045612 |
| 22 | CYP1A2 | cytochrome P450; family 1; subfamily A; polypeptide 2 | 15 | rs12720461 | 15q24.1b | INTRON | 75041351 |
| 23 | CYP1A2 | cytochrome P450; family 1; subfamily A; polypeptide 2 | 15 | rs762551 | 15q24.1b | INTRON | 75041917 |
| 24 | CYP1A2 | cytochrome P450; family 1; subfamily A; polypeptide 2 | 15 | rs2069514 | 15q24.1b | INTERGENIC/UNKNOWN | 75038220 |
| 25 | CYP2A6 | cytochrome P450; family 2; subfamily A; polypeptide 6 | 19 | rs28399433 | 19q13.2b | INTERGENIC/UNKNOWN | 41356379 |
| 26 | CYP2A6 | cytochrome P450; family 2; subfamily A; polypeptide 6 | 19 | rs28399454 | 19q13.2b | MIS-SENSE MUTATION | 41351267 |
| 27 | CYP2A6 | cytochrome P450; family 2; subfamily A; polypeptide 6 | 19 | NULLhCV60731447 | 19q13.2b | INTERGENIC/UNKNOWN | 41354190 |
| 28 | CYP2A6 | cytochrome P450; family 2; subfamily A; polypeptide 6 | 19 | rs1801272 | 19q13.2b | MIS-SENSE MUTATION | 41354533 |
| 29 | CYP2A6 | cytochrome P450; family 2; subfamily A; polypeptide 6 | 19 | rs4986891 | 19q13.2b | INTERGENIC/UNKNOWN | 41354629 |
| 30 | CYP2A13 | cytochrome P450; family 2; subfamily A; polypeptide 13 | 19 | rs72547591 | 19q13.2b | INTERGENIC/UNKNOWN | 41601773 |
| 31 | CYP2A13 | cytochrome P450; family 2; subfamily A; polypeptide 13 | 19 | NULLhCV33845966 | 19q13.2b | INTERGENIC/UNKNOWN | 41597652 |
| 32 | CYP2B6 | cytochrome P450; family 2; subfamily A; polypeptide 7 pseudogene 1;cytochrome P450; family 2; subfamily B; polypeptide 6 | 19 | rs3745274 | 19q13.2b | MIS-SENSE MUTATION | 41512841 |
| 33 | CYP2B6;CYP2A7P1 | cytochrome P450; family 2; subfamily B; polypeptide 6;  cytochrome P450; family 2; subfamily A; polypeptide 7  pseudogene 1 | 19 | rs34097093 | 19q13.2b | NONSENSE MUTATION | 41518370 |
| 34 | CYP2B6;CYP2A7P1 | cytochrome P450; family 2; subfamily A;  polypeptide 7 pseudogene 1;cytochrome P450;  family 2; subfamily B; polypeptide 6 | 19 | rs28399499 | 19q13.2b | MIS-SENSE MUTATION | 41518221 |
| 35 | CYP2B6;CYP2A7P1 | cytochrome P450; family 2; subfamily A;  polypeptide 7 pseudogene 1;cytochrome P450; family 2;  subfamily B; polypeptide 6 | 19 | rs12721655 | 19q13.2b | MIS-SENSE MUTATION | 41510282 |
| 36 | CYP2C8 | cytochrome P450; family 2; subfamily C; polypeptide 8 | 10 | rs72558195 | 10q23.33d | INTERGENIC/UNKNOWN | 96824643 |
| 37 | CYP2C8 | cytochrome P450; family 2; subfamily C; polypeptide 8 | 10 | rs72558196 | 10q23.33d | INSERTION/DELETION | 96826971 |
| 38 | CYP2C8 | cytochrome P450; family 2; subfamily C; polypeptide 8 | 10 | rs1058930 | 10q23.33d | MIS-SENSE MUTATION | 96818119 |
| 39 | CYP2C8 | cytochrome P450; family 2; subfamily C; polypeptide 8 | 10 | rs11572103 | 10q23.33d | MIS-SENSE MUTATION | 96818106 |
| 40 | CYP2C8 | cytochrome P450; family 2; subfamily C; polypeptide 8 | 10 | rs10509681 | 10q23.33d | MIS-SENSE MUTATION | 96798749 |
| 41 | CYP2C8 | cytochrome P450; family 2; subfamily C; polypeptide 8 | 10 | rs11572080 | 10q23.33d | MIS-SENSE MUTATION | 96827030 |
| 42 | CYP2C8 | cytochrome P450; family 2; subfamily C; polypeptide 8 | 10 | rs72558195 | 10q23.33d | INTERGENIC/UNKNOWN | 96824643 |
| 43 | CYP2C9 | cytochrome P450; family 2; subfamily C; polypeptide 9 | 10 | rs1799853 | 10q23.33d | MIS-SENSE MUTATION | 96702047 |
| 44 | CYP2C9 | cytochrome P450; family 2; subfamily C; polypeptide 9 | 10 | rs7900194 | 10q23.33d | MIS-SENSE MUTATION | 96702066 |
| 45 | CYP2C9 | cytochrome P450; family 2; subfamily C; polypeptide 9 | 10 | rs28371686 | 10q23.33d | MIS-SENSE MUTATION | 96741058 |
| 46 | CYP2C9 | cytochrome P450; family 2; subfamily C; polypeptide 9 | 10 | rs7900194 | 10q23.33d | MIS-SENSE MUTATION | 96702066 |
| 47 | CYP2C9 | cytochrome P450; family 2; subfamily C; polypeptide 9 | 10 | rs72558190 | 10q23.33d | NONSENSE MUTATION | 96707539 |
| 48 | CYP2C9 | cytochrome P450; family 2; subfamily C; polypeptide 9 | 10 | rs9332130 | 10q23.33d | MIS-SENSE MUTATION | 96709037 |
| 49 | CYP2C9 | cytochrome P450; family 2; subfamily C; polypeptide 9 | 10 | rs9332239 | 10q23.33d | MIS-SENSE MUTATION | 96748777 |
| 50 | CYP2C9 | cytochrome P450; family 2; subfamily C; polypeptide 9 | 10 | rs1057910 | 10q23.33d | MIS-SENSE MUTATION | 96741053 |
| 51 | CYP2C9 | cytochrome P450; family 2; subfamily C; polypeptide 9 | 10 | rs28371685 | 10q23.33d | MIS-SENSE MUTATION | 96740981 |
| 52 | CYP2C9 | cytochrome P450; family 2; subfamily C; polypeptide 9 | 10 | NULLhCV72649992 | 10q23.33d | INTERGENIC/UNKNOWN | 96701970 |
| 53 | CYP2C9 | cytochrome P450; family 2; subfamily C; polypeptide 9 | 10 | rs72558187 | 10q23.33d | MIS-SENSE MUTATION | 96701715 |
| 54 | CYP2C9 | cytochrome P450; family 2; subfamily C; polypeptide 9 | 10 | rs9332131 | 10q23.33d | INTERGENIC/UNKNOWN | 96709040 |
| 55 | CYP2C19 | cytochrome P450; family 2; subfamily C; polypeptide 19 | 10 | rs17885098 | 10q23.33d | SILENT MUTATION | 96522561 |
| 56 | CYP2C19 | cytochrome P450; family 2; subfamily C; polypeptide 19 | 10 | rs72558186 | 10q23.33d | DONOR SPLICE SITE | 96541756 |
| 57 | CYP2C19 | cytochrome P450; family 2; subfamily C; polypeptide 19 | 10 | rs4986893 | 10q23.33d | NONSENSE MUTATION | 96540410 |
| 58 | CYP2C19 | cytochrome P450; family 2; subfamily C; polypeptide 19 | 10 | rs12248560 | 10q23.33d | INTERGENIC/UNKNOWN | 96521657 |
| 59 | CYP2C19 | cytochrome P450; family 2; subfamily C; polypeptide 19 | 10 | rs17886522 | 10q23.33d | SILENT MUTATION | 96609775 |
| 60 | CYP2C19 | cytochrome P450; family 2; subfamily C; polypeptide 19 | 10 | rs41291556 | 10q23.33d | MIS-SENSE MUTATION | 96535173 |
| 61 | CYP2C19 | cytochrome P450; family 2; subfamily C; polypeptide 19 | 10 | rs3758580 | 10q23.33d | SILENT MUTATION | 96602622 |
| 62 | CYP2C19 | cytochrome P450; family 2; subfamily C; polypeptide 19 | 10 | rs28399504 | 10q23.33d | MIS-SENSE MUTATION | 96522463 |
| 63 | CYP2C19 | cytochrome P450; family 2; subfamily C; polypeptide 19 | 10 | rs28399507 | 10q23.33d | MIS-SENSE MUTATION | 96535246 |
| 64 | CYP2C19 | cytochrome P450; family 2; subfamily C; polypeptide 19 | 10 | rs17878459 | 10q23.33d | MIS-SENSE MUTATION | 96534922 |
| 65 | CYP2C19 | cytochrome P450; family 2; subfamily C; polypeptide 19 | 10 | rs55640102 | 10q23.33d | INTERGENIC/UNKNOWN | 96612671 |
| 66 | CYP2C19 | cytochrome P450; family 2; subfamily C; polypeptide 19 | 10 | rs4244285 | 10q23.33d | INTERGENIC/UNKNOWN | 96541616 |
| 67 | CYP2C19 | cytochrome P450; family 2; subfamily C; polypeptide 19 | 10 | rs56337013 | 10q23.33d | MIS-SENSE MUTATION | 96612495 |
| 68 | CYP2C19 | cytochrome P450; family 2; subfamily C; polypeptide 19 | 10 | rs72552267 | 10q23.33d | MIS-SENSE MUTATION | 96535210 |
| 69 | CYP2D6 | cytochrome P450; family 2; subfamily D; polypeptide 6;  uncharacterized LOC100132273 | 22 | rs5030867 | 22q13.2b | MIS-SENSE MUTATION | 42523858 |
| 70 | CYP2D6 | cytochrome P450; family 2; subfamily D;  polypeptide 7 pseudogene 1;uncharacterized LOC100132273;  cytochrome P450; family 2; subfamily D; polypeptide 6 | 22 | rs5030862 | 22q13.2b | MIS-SENSE MUTATION | 42526670 |
| 71 | CYP2D6 | uncharacterized LOC100132273;cytochrome P450; family 2;  subfamily D; polypeptide 6 | 22 | rs72549351 | 22q13.2b | INSERTION/DELETION | 42524203 |
| 72 | CYP2D6 | uncharacterized LOC100132273;cytochrome P450; family 2;  subfamily D; polypeptide 6 | 22 | NULL hCV32407240 | 22q13.2b | INTERGENIC/UNKNOWN | 42524930 |
| 73 | CYP2D6;LOC100132273 | cytochrome P450; family 2; subfamily D; polypeptide 6;  uncharacterized LOC100132273 | 22 | rs3892097 | 22q13.2b | ACCEPTOR SPLICE SITE | 42524947 |
| 74 | CYP2D6;LOC100132273 | cytochrome P450; family 2; subfamily D; polypeptide 6;  uncharacterized LOC100132273 | 22 | rs5030865 | 22q13.2b | INTERGENIC/UNKNOWN | 42525035 |
| 75 | CYP2D6;LOC100132273 | cytochrome P450; family 2; subfamily D; polypeptide 6;  uncharacterized LOC100132273 | 22 | rs5030865 | 22q13.2b | INTERGENIC/UNKNOWN | 42525035 |
| 76 | CYP2D6;LOC100132273 | cytochrome P450; family 2; subfamily D; polypeptide 6;  uncharacterized LOC100132273 | 22 | rs72549354 | 22q13.2b | INSERTION/DELETION | 42524819 |
| 77 | CYP2D6;LOC100132273 | cytochrome P450; family 2; subfamily D; polypeptide 6;  uncharacterized LOC100132273 | 22 | rs72549346 | 22q13.2b | INSERTION/DELETION | 42523533 |
| 78 | CYP2D6;LOC100132273 | cytochrome P450; family 2; subfamily D; polypeptide 6;  uncharacterized LOC100132273 | 22 | rs147960066 | 22q13.2b | INTERGENIC/UNKNOWN | 42523592 |
| 79 | CYP2D6;LOC100132273 | cytochrome P450; family 2; subfamily D; polypeptide 6;  uncharacterized LOC100132273 | 22 | rs35742686 | 22q13.2b | INSERTION/DELETION | 42524244 |
| 80 | CYP2D6;LOC100132273 | uncharacterized LOC100132273;cytochrome P450; family 2;  subfamily D; polypeptide 6 | 22 | rs72549350 | 22q13.2b | INTERGENIC/UNKNOWN | 42524178 |
| 81 | CYP2D6;LOC100132273 | cytochrome P450; family 2; subfamily D; polypeptide 6;  uncharacterized LOC100132273 | 22 | rs72549353 | 22q13.2b | INTERGENIC/UNKNOWN | 42524251 |
| 82 | CYP2D6;CYP2D7P1;LOC100132273 | cytochrome P450; family 2; subfamily D; polypeptide 6;  cytochrome P450; family 2; subfamily D;  polypeptide 7 pseudogene 1;  uncharacterized LOC100132273 | 22 | rs72549357 | 22q13.2b | INTERGENIC/UNKNOWN | 42526657 |
| 83 | CYP2D6;LOC100132273 | uncharacterized LOC100132273;cytochrome P450; family 2;  subfamily D; polypeptide 6 | 22 | NULL hCV32407220 | 22q13.2b | INTERGENIC/UNKNOWN | 42522669 |
| 84 | CYP2D6;LOC100132273 | cytochrome P450; family 2; subfamily D; polypeptide 6;  uncharacterized LOC100132273 | 22 | rs5030863 | 22q13.2b | INTERGENIC/UNKNOWN | 42525912 |
| 85 | CYP2D6;LOC100132273 | cytochrome P450; family 2; subfamily D; polypeptide 6;  uncharacterized LOC100132273 | 22 | rs72549349 | 22q13.2b | DONOR SPLICE SITE | 42523843 |
| 86 | CYP2D6;LOC100132273 | cytochrome P450; family 2; subfamily D; polypeptide 6;  uncharacterized LOC100132273 | 22 | rs28371706 | 22q13.2b | MIS-SENSE MUTATION | 42525772 |
| 87 | CYP2D6;LOC100132273 | cytochrome P450; family 2; subfamily D; polypeptide 6;  uncharacterized LOC100132273 | 22 | rs5030655 | 22q13.2b | INTRON | 42525086 |
| 88 | CYP2E1 | cytochrome P450; family 2; subfamily E; polypeptide 1 | 10 | rs72559710 | 10q26.3f | INTERGENIC/UNKNOWN | 135342034 |
| 89 | CYP3A4 | cytochrome P450; family 3; subfamily A; polypeptide 4 | 7 | rs4646438 | 7q22.1b | INSERTION/DELETION | 99364034 |
| 90 | CYP3A4 | cytochrome P450; family 3; subfamily A; polypeptide 4 | 7 | rs55785340 | 7q22.1b | MIS-SENSE MUTATION | 99365983 |
| 91 | CYP3A5 | cytochrome P450; family 3; subfamily A; polypeptide 5 | 7 | rs10264272 | 7q22.1b | SILENT MUTATION | 99262835 |
| 92 | CYP3A5 | cytochrome P450; family 3; subfamily A; polypeptide 5 | 7 | rs41279854 | 7q22.1b | MIS-SENSE MUTATION | 99247772 |
| 93 | CYP3A5 | cytochrome P450; family 3; subfamily A; polypeptide 5 | 7 | rs41303343 | 7q22.1b | INSERTION/DELETION | 99250393 |
| 94 | CYP3A5 | cytochrome P450; family 3; subfamily A; polypeptide 5 | 7 | rs55965422 | 7q22.1b | INTERGENIC/UNKNOWN | 99264573 |
| 95 | DPYD | dihydropyrimidine dehydrogenase | 1 | rs1801266 | 1p21.3b | MIS-SENSE MUTATION | 98157332 |
| 96 | DPYD | dihydropyrimidine dehydrogenase | 1 | rs1801268 | 1p21.3b | MIS-SENSE MUTATION | 97544627 |
| 97 | DPYD | dihydropyrimidine dehydrogenase | 1 | NULL hCV32287186 | 1p21.3b | INTERGENIC/UNKNOWN | 98205971 |
| 98 | DPYD | dihydropyrimidine dehydrogenase | 1 | rs1801267 | 1p21.3b | MIS-SENSE MUTATION | 97564154 |
| 99 | DPYD | dihydropyrimidine dehydrogenase | 1 | rs1801265 | 1p21.3b | MIS-SENSE MUTATION | 98348885 |
| 100 | DPYD | dihydropyrimidine dehydrogenase | 1 | rs3918290 | 1p21.3b | DONOR SPLICE SITE | 97915614 |
| 101 | GSTP1 | glutathione S-transferase pi 1 | 11 | rs1695 | 11q13.2b | MIS-SENSE MUTATION | 67352689 |
| 102 | NAT1 | N-acetyltransferase 1 (arylamine N-acetyltransferase) | 8 | rs55793712 | 8p22a | INTERGENIC/UNKNOWN | 18080440 |
| 103 | NAT1 | N-acetyltransferase 1 (arylamine N-acetyltransferase) | 8 | rs56172717 | 8p22a | MIS-SENSE MUTATION | 18080308 |
| 104 | NAT1 | N-acetyltransferase 1 (arylamine N-acetyltransferase) | 8 | rs4986782 | 8p22a | MIS-SENSE MUTATION | 18080116 |
| 105 | NAT1 | N-acetyltransferase 1 (arylamine N-acetyltransferase) | 8 | rs56318881 | 8p22a | NONSENSE MUTATION | 18079653 |
| 106 | NAT1 | N-acetyltransferase 1 (arylamine N-acetyltransferase) | 8 | rs56379106 | 8p22a | MIS-SENSE MUTATION | 18079746 |
| 107 | NAT1 | N-acetyltransferase 1 (arylamine N-acetyltransferase) | 8 | rs5030839 | 8p22a | NONSENSE MUTATION | 18080115 |
| 108 | NAT1 | N-acetyltransferase 1 (arylamine N-acetyltransferase) | 8 | rs4986988 | 8p22a | INTRON | 18079213 |
| 109 | NAT2 | N-acetyltransferase 2 (arylamine N-acetyltransferase) | 8 | rs1805158 | 8p22a | MIS-SENSE MUTATION | 18257703 |
| 110 | NAT2 | N-acetyltransferase 2 (arylamine N-acetyltransferase) | 8 | rs1208 | 8p22a | MIS-SENSE MUTATION | 18258316 |
| 111 | NAT2 | N-acetyltransferase 2 (arylamine N-acetyltransferase) | 8 | rs1041983 | 8p22a | SILENT MUTATION | 18257795 |
| 112 | NAT2 | N-acetyltransferase 2 (arylamine N-acetyltransferase) | 8 | rs1799929 | 8p22a | SILENT MUTATION | 18257994 |
| 113 | NAT2 | N-acetyltransferase 2 (arylamine N-acetyltransferase) | 8 | rs1799931 | 8p22a | MIS-SENSE MUTATION | 18258370 |
| 114 | NAT2 | N-acetyltransferase 2 (arylamine N-acetyltransferase) | 8 | rs1801279 | 8p22a | MIS-SENSE MUTATION | 18257704 |
| 115 | NAT2 | N-acetyltransferase 2 (arylamine N-acetyltransferase) | 8 | rs1799930 | 8p22a | MIS-SENSE MUTATION | 18258103 |
| 116 | NAT2 | N-acetyltransferase 2 (arylamine N-acetyltransferase) | 8 | rs1801280 | 8p22a | MIS-SENSE MUTATION | 18257854 |
| 117 | SLC15A2 | solute carrier family 15 (H+/peptide transporter); member 2 | 3 | rs1143672 | 3q13.33c | MIS-SENSE MUTATION | 121648168 |
| 118 | SLC15A2 | solute carrier family 15 (H+/peptide transporter); member 2 | 3 | rs2293616 | 3q13.33c | SILENT MUTATION | 121641693 |
| 119 | SLC15A2 | solute carrier family 15 (H+/peptide transporter); member 2 | 3 | rs2257212 | 3q13.33c | MIS-SENSE MUTATION | 121643804 |
| 120 | SLC15A2 | solute carrier family 15 (H+/peptide transporter); member 2 | 3 | rs1143671 | 3q13.33c | MIS-SENSE MUTATION | 121647286 |
| 121 | SLC22A1 | solute carrier family 22 (organic cation transporter); member 1 | 6 | rs2282143 | 6q25.3f | MIS-SENSE MUTATION | 160557643 |
| 122 | SLC22A1 | solute carrier family 22 (organic cation transporter); member 1 | 6 | rs72552763 | 6q25.3f | INSERTION/DELETION | 160560883 |
| 123 | SLC22A1 | solute carrier family 22 (organic cation transporter); member 1 | 6 | rs628031 | 6q25.3f | MIS-SENSE MUTATION | 160560845 |
| 124 | SLC22A1 | solute carrier family 22 (organic cation transporter); member 1 | 6 | rs34059508 | 6q25.3f | INTERGENIC/UNKNOWN | 160575837 |
| 125 | SLC22A1 | solute carrier family 22 (organic cation transporter); member 1 | 6 | rs36103319 | 6q25.3f | INTERGENIC/UNKNOWN | 160553407 |
| 126 | SLC22A1 | solute carrier family 22 (organic cation transporter); member 1 | 6 | rs55918055 | 6q25.3f | INTERGENIC/UNKNOWN | 160543229 |
| 127 | SLC22A1 | solute carrier family 22 (organic cation transporter); member 1 | 6 | rs4646278 | 6q25.3f | MIS-SENSE MUTATION | 160557271 |
| 128 | SLC22A1 | solute carrier family 22 (organic cation transporter); member 1 | 6 | rs4646277 | 6q25.3f | MIS-SENSE MUTATION | 160557260 |
| 129 | SLC22A2 | solute carrier family 22 (organic cation transporter); member 2 | 6 | rs8177507 | 6q25.3f | INTERGENIC/UNKNOWN | 160677669 |
| 130 | SLC22A2 | solute carrier family 22 (organic cation transporter); member 2 | 6 | rs8177517 | 6q25.3f | MIS-SENSE MUTATION | 160663420 |
| 131 | SLC22A2 | solute carrier family 22 (organic cation transporter); member 2 | 6 | rs8177504 | 6q25.3f | MIS-SENSE MUTATION | 160679630 |
| 132 | SLC22A2 | solute carrier family 22 (organic cation transporter); member 2 | 6 | rs316019 | 6q25.3f | MIS-SENSE MUTATION | 160670282 |
| 133 | SLC22A2 | solute carrier family 22 (organic cation transporter); member 2 | 6 | rs8177516 | 6q25.3f | MIS-SENSE MUTATION | 160664685 |
| 134 | SLC22A6;SLC22A8 | solute carrier family 22 (organic anion transporter);  member 6;solute carrier family 22 (organic anion transporter);  member 8 | 11 | rs11568626 | 11q12.3b | MIS-SENSE MUTATION | 62752014 |
| 135 | SLCO2B1 | solute carrier organic anion transporter family; member 2B1 | 11 | rs2306168 | 11q13.4c | MIS-SENSE MUTATION | 74907582 |
| 136 | SLCO1B1 | solute carrier organic anion transporter family; member 1B1 | 12 | rs59502379 | 12p12.1e | MIS-SENSE MUTATION | 21358933 |
| 137 | SLCO1B1 | solute carrier organic anion transporter family; member 1B1 | 12 | rs2306283 | 12p12.1e | MIS-SENSE MUTATION | 21329738 |
| 138 | SLCO1B1 | solute carrier organic anion transporter family; member 1B1 | 12 | rs72559745 | 12p12.1e | MIS-SENSE MUTATION | 21329817 |
| 139 | SLCO1B1 | solute carrier organic anion transporter family; member 1B1 | 12 | rs55737008 | 12p12.1e | MIS-SENSE MUTATION | 21392047 |
| 140 | SLCO1B1 | solute carrier organic anion transporter family; member 1B1 | 12 | rs4149056 | 12p12.1e | MIS-SENSE MUTATION | 21331549 |
| 141 | SLCO1B1 | solute carrier organic anion transporter family; member 1B1 | 12 | rs56061388 | 12p12.1e | MIS-SENSE MUTATION | 21327529 |
| 142 | SLCO1B1 | solute carrier organic anion transporter family; member 1B1 | 12 | rs56101265 | 12p12.1e | MIS-SENSE MUTATION | 21325716 |
| 143 | SLCO1B3 | solute carrier organic anion transporter family; member 1B3 | 12 | rs7311358 | 12p12.2a | INTERGENIC/UNKNOWN | 21015760 |
| 144 | SLCO1B3 | solute carrier organic anion transporter family; member 1B3 | 12 | rs4149117 | 12p12.2a | INTERGENIC/UNKNOWN | 21011480 |
| 145 | TPMT | thiopurine S-methyltransferase | 6 | rs1800460 | 6p22.3e | MIS-SENSE MUTATION | 18139228 |
| 146 | TPMT | thiopurine S-methyltransferase | 6 | rs1800462 | 6p22.3e | MIS-SENSE MUTATION | 18143955 |
| 147 | NHLRC1;TPMT | NHL repeat containing 1;thiopurine S-methyltransferase | 6 | rs1800584 | 6p22.3e | ACCEPTOR SPLICE SITE | 18131012 |
| 148 | NHLRC1;TPMT | NHL repeat containing 1;thiopurine S-methyltransferase | 6 | rs1142345 | 6p22.3e | MIS-SENSE MUTATION | 18130918 |
| 149 | NHLRC1;TPMT | NHL repeat containing 1;thiopurine S-methyltransferase | 6 | rs56161402 | 6p22.3e | MIS-SENSE MUTATION | 18130993 |
| 150 | UGT1A3 | UDP glucuronosyltransferase 1 family; polypeptide A3;  DnaJ (Hsp40) homolog; subfamily B; member 3 pseudogene;  UDP glucuronosyltransferase 1 family; polypeptide A4;  UDP glucuronosyltransferase 1 family; polypeptide A7;  UDP glucuronosyltransferase 1 family; pol | 2 | rs35350960 | 2q37.1d | INTERGENIC/UNKNOWN | 234669619 |
| 151 | UGT1A4 | UDP glucuronosyltransferase 1 family; polypeptide A4;  UDP glucuronosyltransferase 1 family; polypeptide A6;  DnaJ (Hsp40) homolog; subfamily B; member 3 pseudogene;  UDP glucuronosyltransferase 1 family; polypeptide A9;  UDP glucuronosyltransferase 1 family; pol | 2 | rs4148323 | 2q37.1d | INTRON | 234669144 |
| 152 | UGT1A7 | UDP glucuronosyltransferase 1 family; polypeptide A7;  UDP glucuronosyltransferase 1 family; polypeptide A6;  UDP glucuronosyltransferase 1 family; polypeptide A4;  UDP glucuronosyltransferase 1 family; polypeptide A5;  UDP glucuronosyltransferase 1 family; polyp | 2 | rs4124874 | 2q37.1d | INTRON | 234665659 |
| 153 | UGT1A8 | UDP glucuronosyltransferase 1 family; polypeptide A8;  UDP glucuronosyltransferase 1 family; polypeptide A3;  UDP glucuronosyltransferase 1 family; polypeptide A9;  UDP glucuronosyltransferase 1 family; polypeptide A5;  UDP glucuronosyltransferase 1 family; polyp | 2 | rs55750087 | 2q37.1d | MIS-SENSE MUTATION | 234676880 |
| 154 | UGT1A9; | UDP glucuronosyltransferase 1 family; polypeptide A9;  UDP glucuronosyltransferase 1 family; polypeptide A5;  HEAT repeat containing 7B1;  UDP glucuronosyltransferase 1 family; polypeptide A4;  UDP glucuronosyltransferase 1 family; polypeptide A8;  UDP glucuronosyl | 2 | rs34993780 | 2q37.1e | INTERGENIC/UNKNOWN | 234681059 |
| 155 | UGT2B7 | UDP glucuronosyltransferase 2 family; polypeptide B7 | 4 | rs7668258 | 4q13.2c | INTERGENIC/UNKNOWN | 69962078 |
| 156 | UGT2B7 | UDP glucuronosyltransferase 2 family; polypeptide B7 | 4 | rs7662029 | 4q13.2c | INTERGENIC/UNKNOWN | 69961912 |
| 157 | UGT2B15 | UDP glucuronosyltransferase 2 family; polypeptide B15 | 4 | rs1902023 | 4q13.2c | INTERGENIC/UNKNOWN | 69536084 |
| 158 | VKORC1 | protease; serine; 53;vitamin K epoxide reductase complex;  subunit 1;zinc finger protein 646 | 16 | rs8050894 | 16p11.2c | Intron | 31104509 |
