# Supplementary material for: Polymorphisms in genes involved in the absorption, distribution, metabolism, and excretion of drugs in the Kazakhs of Kazakhstan
Source: BMC Genet. 2016 Jan 19;17:23. doi: 10.1186/s12863-016-0329-x (PMC4717528; doi:10.1186/s12863-016-0329-x)
Supplement: Additional file 2: — A list of SNPs that were not found in heterozygous or homozygous variants. (DOC 83 kb) [file 12863_2016_329_MOESM2_ESM.doc]

**Additional file 2.** A list of SNPs that were not found in heterozygous or homozygous variants.

| # | **Assay name** | **RS** |
| --- | --- | --- |
| 1 | CYP2C9*10 g.10598A>G | rs9332130 |
| 2 | CYP1A1*7 g.2346_2347insT | rs72547510 |
| 3 | CYP2D6*38 g.2587-2590delGACT | rs72549351 |
| 4 | NAT1*19_97C>T | rs56318881 |
| 5 | CYP2C19*6 g.12748G>A R132Q | rs72552267 |
| 6 | CYP2C19*5 g.90033C>T | rs56337013 |
| 7 | CYP2D6*12 g.124G>A | rs5030862 |
| 8 | CYP2A6*11 g.3391T>C | hCV33845966 |
| 9 | TPMT*4A_626-1G>A | rs1800584 |
| 10 | NAT1*17_190C>T | rs56379106 |
| 11 | CYP3A5*7 g.27131_27132insT | rs41303343 |
| 12 | CYP2D6*11 g.883G>C | rs5030863 |
| 13 | CYP2C8*5 g.2189delA | rs72558196 |
| 14 | NAT1*15_559C>T | rs5030839 |
| 15 | CYP2C19*4 g.1A>G | rs28399504 |
| 16 | UGT1A1*29_1099C>G | rs55750087 |
| 17 | CYP2C19*12 g.90209A>C | rs55640102 |
| 18 | DPYD*10_V995F | rs1801268 |
| 19 | NAT2*14_191G>A | rs1801279 |
| 20 | CYP2D6*19 g.2539_2542delAACT | rs72549353 |
| 21 | UGT1A1*7_1456T>G | rs34993780 |
| 22 | CYP2C9*6 g.10601delA | rs9332131 |
| 23 | CYP2C9*25 g.3531_3540delAGAAATGGAA | hCV72649992 |
| 24 | ABCG2_Q126X | rs72552713 |
| 25 | DPYD*8_R235W | rs1801266 |
| 26 | CYP2C9*8 g.3627G>A | rs7900194 |
| 27 | CYP2D6*15 g.137-138insT | rs72549357 |
| 28 | SLCO1B1*3_*13_E156G | rs72559745 |
| 29 | CYP2D6*18 g.4125_4133dupGTGCCCACT | hCV32407220 |
| 30 | SLCO1B1*11_E667G | rs55737008 |
| 31 | CYP2C9*13 g.3276T>C | rs72558187 |
| 32 | SLC22A6_R50H | rs11568626 |
| 33 | CYP2A6*6 g.1703G>A | rs4986891 |
| 34 | CYP1A1*6 g.1636G>T | rs56313657 |
| 35 | CYP1A1*8 g.2414T>A | rs72547509 |
| 36 | SLC22A2*7.1_R400C | rs8177516 |
| 37 | ABCC2_R768W | rs56199535 |
| 38 | SLC22A1_R287G | rs4646278 |
| 39 | CYP2D6*20 g.1973_1974insG | rs72549354 |
| 40 | CYP2C9*15 g.9100C>A | rs72558190 |
| 41 | CYP2C9*5 g.42619C>G | rs28371686 |
| 42 | CYP2C8*8 g.4517C>G | rs72558195 |
| 43 | CYP2D6*14 g.1758G>A | rs5030865 |
| 44 | CYP2D6*17 g.1023C>T | rs28371706 |
| 45 | CYP1A1*3 g.3205T>C | rs1800031 |
| 46 | CYP2D6*44 g.2950G>C | rs72549349 |
| 47 | CYP2C19 | rs28399507 |
| 48 | SLCO1B1*3_*13_V82A | rs56061388 |
| 49 | SLCO1B1 | rs56101265 |
| 50 | CYP1A2*7 g.3533G>A | rs56107638 |
| 51 | TPMT*8_R215H | rs56161402 |
| 52 | NAT1*22_752A>T | rs56172717 |
| 53 | SLC22A1_G220V | rs36103319 |
| 54 | ABCC2_S789F | rs56220353 |
| 55 | SLC22A1_C88R | rs55918055 |
| 56 | CYP2D6*42 g.3259_3260insGT | rs72549346 |
| 57 | CYP2D6*56 g.3201C>T | rs147960066 |
| 58 | CYP2C8*7 g.4517C>T | rs72558195 |
| 59 | CYP2C9*27 g.3627G>T | rs7900194 |
| 60 | CYP2B6*16 g. 21011T>C | rs28399499 |
| 61 | CYP2D6*8 g.1758G>T | rs5030865 |
| 62 | CYP2D6*6 g.1707delT | rs5030655 |
| 63 | CYP3A5*10 g.29753T>C | rs41279854 |
| 64 | ABCC2_A1450T | rs56296335 |
| 65 | SLCO1B1*9_G488A | rs59502379 |
| 66 | CYP2B6*28 g.21160C>T | rs34097093 |
| 67 | SLC22A2_P54S | rs8177504 |
| 68 | CYP2A6*7 g.6558T>C | rs72547591 |
| 69 | CYP2A6*17 g.5065G>A | rs28399454 |
| 70 | TPMT*2_A80P | rs1800462 |
| 71 | CYP3A5*6 g.14690G>A | rs10264272 |
| 72 | CYP2C19*7 g.19294T>A | rs72558186 |
| 73 | NAT2*19_190C>T | rs1805158 |
| 74 | DPYD*9B_R886H | rs1801267 |
| 75 | CYP2A6*20 g.2141_2142delAA | hCV60731447 |
