# Supplementary material for: Polymorphisms in genes involved in the absorption, distribution, metabolism, and excretion of drugs in the Kazakhs of Kazakhstan
Source: BMC Genet. 2016 Jan 19;17:23. doi: 10.1186/s12863-016-0329-x (PMC4717528; doi:10.1186/s12863-016-0329-x)
Supplement: Additional file 3: — LD SNP plot. LD analysis of the SLC15A2, UGT2B7, NAT2, and SLCO1B3 genes in 11 populations (HapMap data) and the Kazakh population (our data). A. SLC15A2, B. UGT2B7, C. NAT2, D. SLCO1B3. (DOC 2457 kb) [file 12863_2016_329_MOESM3_ESM.doc]

***Additional file 3.*** *LD SNP plot. LD analysis of the SLC15A2, UGT2B7, NAT2, and SLCO1B3 genes in 11 populations (HapMap data) and the Kazakh population (our data).* ***A.*** *SLC15A2,* ***B.*** *UGT2B7,* ***C.*** *NAT2,* ***D.*** *SLCO1B3.*

| A   |  | | --- | |  |   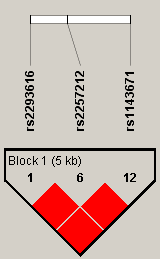 | 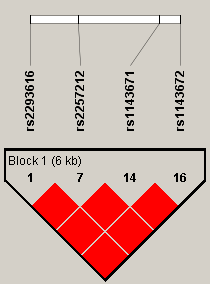 | 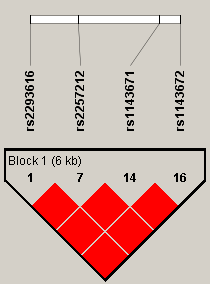 |
| --- | --- | --- | --- | --- |
| **ASW** | **CEU** | **CHB** |
| 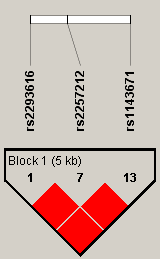 | 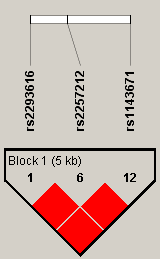 | 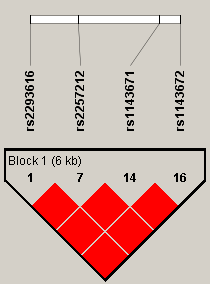 |
| **CHD** | **GIH** | JPT |
| | **A** | | --- |   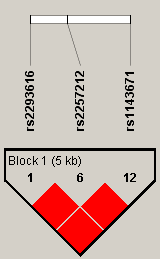 | 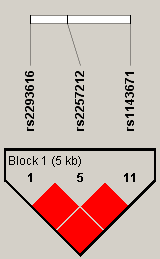 | 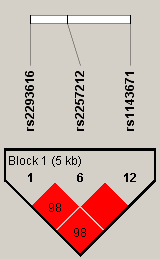 |
| **LWK** | **MEX** | **MKK** |
| 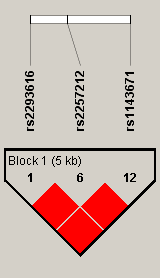 | 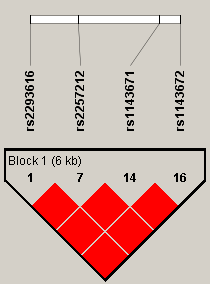 | 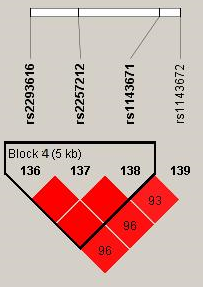 |
| **TSI** | **YRI** | **KAZ** |

| B   |  | | --- | |  |   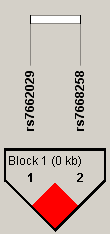 | 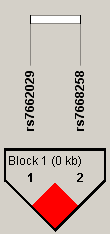 | 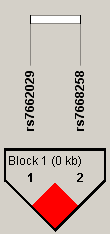 |
| --- | --- | --- | --- | --- |
| **ASW** | **CEU** | **CHB** |
| 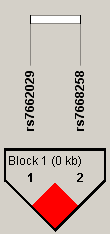 | 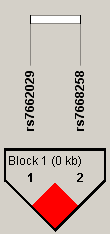 | 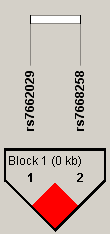 |
| **CHD** | **GIH** | **JPT** |
| 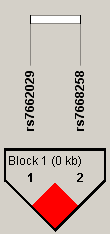 | 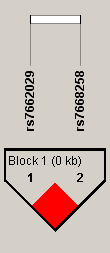 | 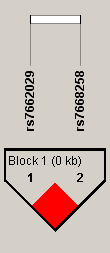 |
| **LWK** | **MEX** | **MKK** |
| 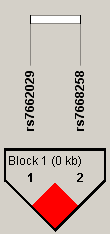 | 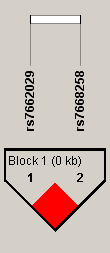 | 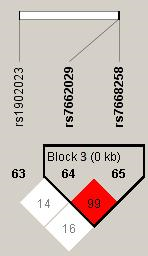 |
| **TSI** | **YRI** | **KAZ** |

| C   |  | | --- | |  |   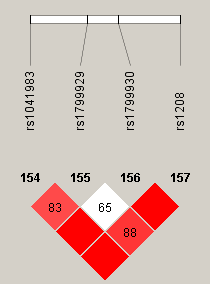 | 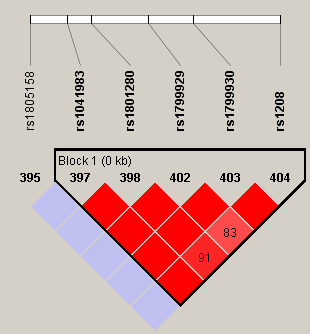 | 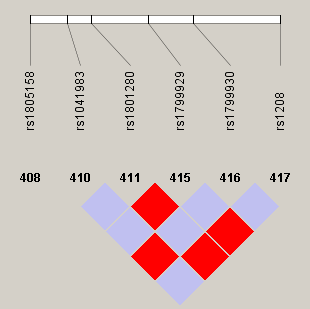 |
| --- | --- | --- | --- | --- |
| **ASW** | **CEU** | **CHB** |
| 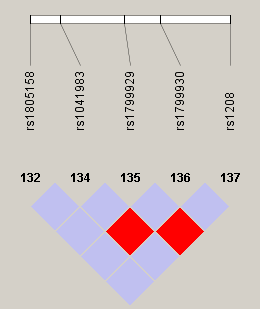 | 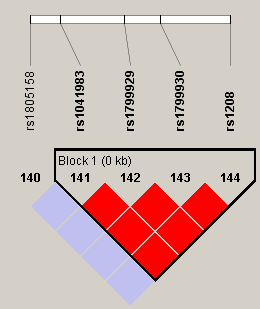 | 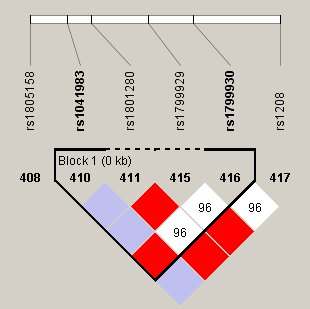 |
| **CHD** | **GIH** | JPT |
| | **C** | | --- |   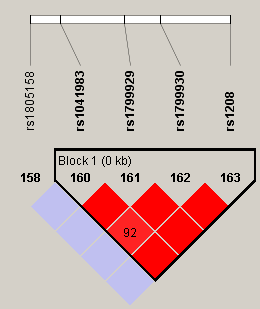 | 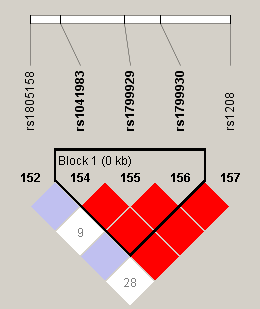 | 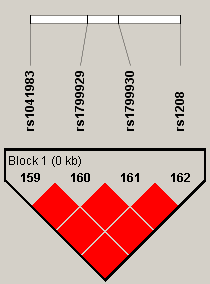 |
| **LWK** | **MEX** | **MKK** |
| 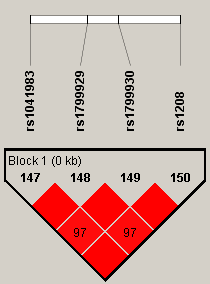 | 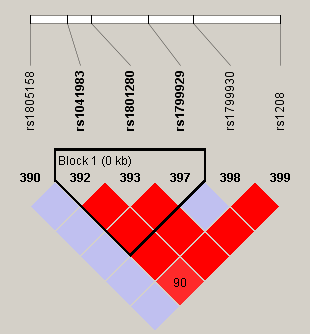 | 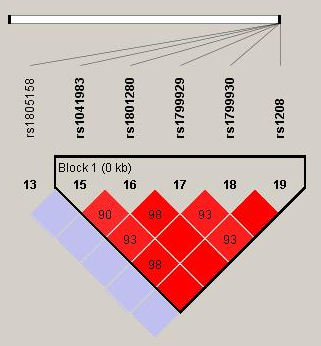 |
| **TSI** | **YRI** | **KAZ** |

| D   |  | | --- | |  |   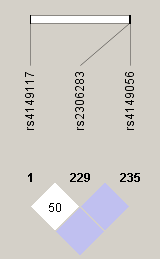 | 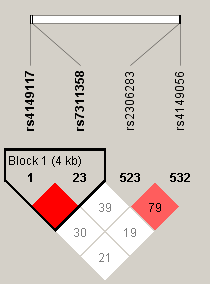 | 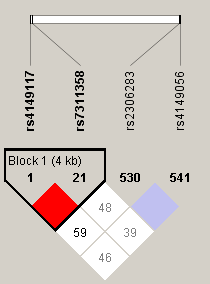 |
| --- | --- | --- | --- | --- |
| **ASW** | **CEU** | **CHB** |
| 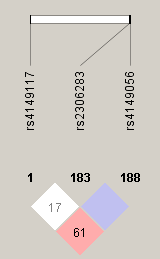 | 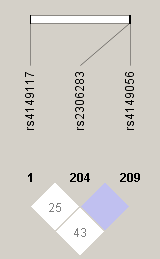 | 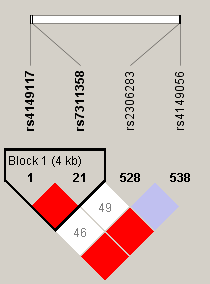 |
| **CHD** | **GIH** | JPT |
| | **D** | | --- |   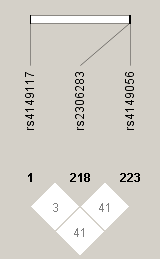 | 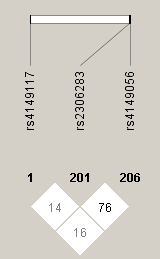 | 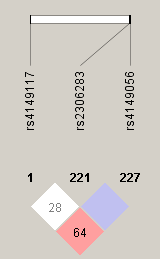 |
| **LWK** | **MEX** | **MKK** |
| 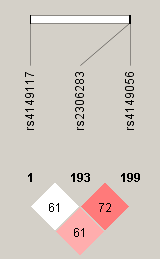 | 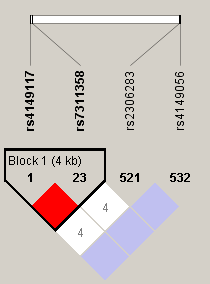 | 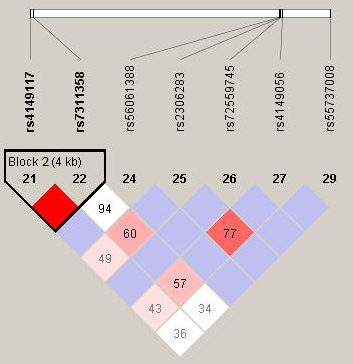 |
| **TSI** | **YRI** | **KAZ** |
